# Exploring *In situ* neuroprotective mechanisms of nicotine in an MPTP-Induced Parkinson’s disease rat model using spatial metabolomics

**DOI:** 10.3389/fcell.2026.1818201

**Published:** 2026-05-01

**Authors:** Lutao Xu, Qian Li, Mingyu Zhu, Gaoge Wang, Hongjuan Wang, Shulei Han, Yaning Fu, Huan Chen, Hongwei Hou

**Affiliations:** 1 Beijing Life Science Academy, Beijing, China; 2 Key Laboratory of Tobacco Biological Effects, China National Tobacco Quality Supervision & Test Center, Zhengzhou, China

**Keywords:** mass spectrometry imaging, multi-brain regions, nicotine, Parkinson disease, spatial metabolomics

## Abstract

Parkinson’s disease (PD) is characterized by profound metabolic disturbances in the brain, yet how these alterations vary across brain regions and respond to neuroprotective intervention remains poorly understood. Here, we applied airflow-assisted desorption electrospray ionization mass spectrometry imaging (AFADESI-MSI) to map spatial metabolic changes in whole brains from 1-methyl-4-phenyl-1,2,3,6-tetrahydropyridine (MPTP)-induced PD rats and to define the *in situ* metabolic effects of nicotine. Spatial metabolomics revealed marked region-dependent metabolic disruption in the PD brain and showed that nicotine broadly reversed the MPTP-induced shift in metabolic trajectories. In particular, nicotine restored dopamine-related metabolites in the striatum and midbrain, normalized γ-aminobutyric acid and serotonin in the hippocampus and thalamus, and alleviated biochemical features of mitochondrial stress by reducing AMP accumulation and restoring glutathione levels. Pathway analysis further identified glycerophospholipid metabolism as a shared regulatory axis across multiple brain regions, suggesting that recovery of membrane lipid homeostasis is a central component of nicotine-mediated neuroprotection. Together, these findings indicate that nicotine exerts spatially coordinated neuroprotective effects in PD by remodeling neurotransmitter balance, improving redox and energy homeostasis, and repairing membrane lipid metabolism. More broadly, this study highlights the value of spatial metabolomics for revealing brain-region-specific metabolic mechanisms of neurodegeneration and drug action.

## 1 Introduction

Parkinson’s disease (PD) is a neurodegenerative disorder that causes severe neurological dysfunction, substantially reduces quality of life, and increases mortality risk (Dickson, 2025; Kurth and Brinks, 2025). Despite extensive investigation, effective strategies for PD prevention and treatment remain limited because of the disease’s complex pathophysiology and pronounced spatial metabolic disturbances within brain tissue (Irmady and Przedborski, 2025). The central nervous system (CNS) has a highly organized and interconnected architecture composed of multiple interacting microregions. Because these brain microregions differ in neural structure, functional roles, and connectivity patterns, endogenous metabolites display strong regional specificity (Kettunen et al., 2024). Accordingly, a detailed understanding of microregion-specific metabolic features and tissue-level metabolic dysregulation is essential for elucidating PD mechanisms and for guiding the development of effective therapeutic interventions (Szunyogh et al., 2025).

Metabolomics offers powerful insights for the systematic investigation of neurological diseases. Researchers have widely applied metabolomic analyses of biological fluids and tissues to elucidate the metabolic mechanisms that drive PD progression and treatment responses (Chan et al., 2025). However, during the complex spatiotemporal cascade of PD, metabolic abnormalities detected in body fluids or whole-brain tissue homogenates may fail to accurately represent pathological changes within specific tissue microregions, particularly in brain tissue protected by the blood–brain barrier (Lau et al., 2024). This limitation is especially important in PD, which is increasingly recognized as a disorder involving anatomically and functionally distinct brain circuits rather than a uniformly affected tissue. Accurately characterizing *in situ* metabolic regulation in the brain remains highly challenging because of its complex architecture and diverse functional microregions. Conventional metabolomic approaches, such as liquid chromatography–mass spectrometry (LC-MS), show clear limitations in the analysis of brain microregions. LC-MS typically requires extensive sample pretreatment, including extraction and homogenization, which inevitably destroys spatial information and can cause metabolite loss or chemical alteration (Mwalembe et al., 2025). Moreover, traditional LC-MS analyses generally report only average metabolite levels across entire tissue samples and therefore lack the spatial resolution needed to distinguish metabolic differences among distinct brain microregions. As a result, although conventional metabolomics can identify altered pathways, it often cannot determine where these changes occur within the brain or how they relate to local neurodegenerative processes. This limitation is particularly relevant in PD research, where metabolic disturbances can differ markedly between regions such as the striatum, hippocampus, and thalamus (Wheeler et al., 2024; Fangma et al., 2023; Neumann, 2025; Vicari et al., 2024; Jha et al., 2024).

Aerodynamic-assisted desorption electrospray ionization mass spectrometry imaging (AFADESI-MSI) is an advanced analytical technology that offers clear advantages for investigating metabolism in brain microregions (Zhu et al., 2022). AFADESI-MSI requires minimal sample pretreatment, which helps preserve endogenous metabolites and their native spatial distributions within tissue sections (He et al., 2025; Spears et al., 2025; Hu et al., 2023). By reducing metabolite loss and chemical alteration, this approach enables more accurate *in situ* metabolic characterization. AFADESI-MSI also provides high spatial resolution (50–100 μm), allowing direct visualization of metabolic alterations at the microregional scale (Zhou et al., 2022; Wang Z. et al., 2023). This capability is particularly valuable in PD research because it enables metabolite changes to be interpreted in their anatomical context and helps reveal how different brain regions respond unequally to neurodegeneration and pharmacological intervention (Yang et al., 2020; Jiang et al., 2023; Kaya et al., 2023a; Shariatgorji et al., 2019; Fridjonsdottir et al., 2021; Zheng et al., 2025; Li et al., 2025; Ma et al., 2024; Lu et al., 2025; Yang et al., 2025; Cheng et al., 2025). By directly mapping small-molecule metabolites, lipids, and drug-related components in tissue sections, AFADESI-MSI delivers a comprehensive view of metabolic remodeling and can reveal pathways and molecular interactions that conventional methods may overlook (Luo et al., 2013). Previous studies have successfully applied MSI-based spatial metabolomics to investigate region-specific metabolic and neurochemical remodeling in neurological diseases and drug-response studies. For example, this technology has been used to elucidate the pharmacological mechanisms by which *Polygala tenuifolia* regulates intracerebral neurochemical networks (Li et al., 2024). In addition, in models of cerebral ischemia–reperfusion injury, mass spectrometry imaging approaches have precisely defined the spatially specific mechanisms by which the Radix Astragali–Safflower herb pair repairs lipid and energy metabolism disorders through visualization of metabolic reprogramming in microregions such as the cerebral cortex and hippocampus (Zhao et al., 2024). Emerging studies in neurodegenerative diseases further support the value of spatial metabolomics for linking metabolic abnormalities to specific anatomical regions and pathological processes (Vallianatou et al., 2023; Kaya et al., 2023b). Together, these studies demonstrate that MSI technology overcomes the spatial limitations of traditional metabolomic methods and provides essential technical support for detailed analysis of microregion-specific metabolic mechanisms in complex neurological diseases, including PD (Yang et al., 2020; Kaya et al., 2023a; Li et al., 2025; Ma et al., 2024; Lu et al., 2025; Yang et al., 2025; Cheng et al., 2025; Hunter et al., 2021; Hunter et al., 2018).

Nicotine, the principal alkaloid in tobacco, shows a consistent negative association with the risk of PD incidence in epidemiological studies (Wang X. et al., 2023). As an agonist of nicotinic acetylcholine receptors (nAChRs), nicotine has been reported to influence neurotransmitter release, synaptic plasticity, inflammatory responses, and neuronal survival, and it exhibits neurobiological effects in multiple PD models (Bono et al., 2019; Stern, 2021). However, nicotine is pharmacologically complex and should not be regarded as a straightforward therapeutic agent. Its effects may depend on dose, route of administration, duration of exposure, receptor subtype engagement, and disease context. Therefore, although nicotine-related signaling may exert potentially beneficial effects under certain experimental conditions, the mechanisms underlying these effects remain insufficiently understood (Lin et al., 2025). In particular, current studies provide limited insight into the metabolic regulatory mechanisms of nicotine in PD, especially its *in situ* effects at the level of brain microregions. This knowledge gap restricts a mechanistic understanding of how nicotine-related signaling reshapes the diseased brain and limits the interpretation of its potential translational relevance (Mwalembe et al., 2025). Most previous investigations of PD therapeutics have relied on LC-MS–based analyses of metabolic alterations in serum or homogenized tissues, whereas direct evidence of metabolic remodeling within distinct brain microregions remains scarce (Wang X. et al., 2023; Wang et al., 2024).

Accordingly, this study applies AFADESI-MSI–based spatial metabolomics to comprehensively characterize metabolic expression patterns and spatial heterogeneity in the 1-methyl-4-phenyl-1,2,3,6-tetrahydropyridine (MPTP)-induced PD rat model. We aim to identify key metabolites and metabolic pathways involved in PD pathogenesis and to define their region-specific distributions across the brain. We further investigate whether and how nicotine modulates these spatial metabolic alterations to alleviate model-induced damage, thereby clarifying the neuroprotective mechanism of nicotine from a spatially resolved metabolic perspective.

## 2 Materials and methods

### 2.1 Drugs and reagents

We purchased MPTP (1-methyl-4-phenyl-1,2,3,6-tetrahydropyridine) hydrochloride from MCE (Monmouth Junction, NJ, United States of America). We synthesized nicotine in-house and confirmed its structure and purity (>99%) by nuclear magnetic resonance (NMR) and mass spectrometry. We obtained chromatographic-grade methanol, acetonitrile, and formic acid from Thermo Fisher Scientific (Shanghai, China). We prepared deionized water using a Milli-Q Academic ultrapure water system (Millipore, Bedford, United States of America). We purchased the embedding agent (CMC-Na) and all other conventional reagents as analytical-grade chemicals.

### 2.2 Animal grouping and model establishment

We obtained male Sprague–Dawley (SD) rats (280 ± 25 g) from Vital River (License No.: CTQTC-SYXK-2025001). We housed the animals under specific pathogen-free (SPF) conditions at 24 °C ± 2 °C and 60% ± 5% humidity with a 12 h light/dark cycle and provided food and water *ad libitum*. After a 5-day acclimation period, we randomly assigned the rats to three groups(n = 6): Control, MPTP, and Nicotine treatment. Only male rats were used in this study to reduce variability associated with sex hormone cycling in behavioral and neurochemical readouts and to maintain consistency in this initial mechanism-focused evaluation.

We induced the PD model in the MPTP and Nicotine treatment groups by intraperitoneal injection of MPTP (30 mg/kg, once daily for 7 consecutive days), with two additional booster injections on day 20 and day 27 to maintain the pathological state (Mustapha and Taib, 2021; Meredith and Rademacher, 2011; Smeyne and Jackson-Lewis, 2005; Jackson-Lewis and Przedborski, 2007; Klemann et al., 2016; AlShimemeri et al., 2022; Wichmann and DeLong, 2003). We administered an equal volume of normal saline to the Control group. The Control group received an equal volume of normal saline, which was also used as the vehicle for the Control and MPTP groups during the nicotine treatment phase.

During or after model induction, we treated the Nicotine group with nicotine by intraperitoneal injection (0.2 mg/kg, once daily for 14 consecutive days), while the Control and MPTP groups received an equal volume of vehicle. The overall experimental procedure lasted 34 days, including 5 days of acclimation, 7 days of model induction, 14 days of intervention, and the subsequent behavioral testing/tissue collection period.

All six rats in each group underwent behavioral testing. After completion of the behavioral assessments, brains were collected for downstream analyses. Of the six animals per group, the striatum regions from three brains per group were used for LC-MS/MS analysis, and the remaining three brains were used for AFADESI-MSI and Western blot (WB) validation. For the latter, the right hemisphere was used for MSI imaging and the left striatal tissue was used for WB analysis. This design allowed behavioral phenotyping and orthogonal biochemical validation to be obtained from the same experimental cohort while minimizing animal use. The Laboratory Animal Management and Ethics Committee of the China National Tobacco Quality Supervision and Test Center approved all animal procedures (Approval No.: CTQTC-SYXK-2025001).

### 2.3 Behavioral assessment

Behavioral tests were performed 24 h after the final drug administration to evaluate motor function, anxiety-like behavior, and spatial working memory. All tests were conducted in a quiet, dimly lit room between 09:00 and 15:00.

#### 2.3.1 Open field test (OFT)

The OFT was used to assess spontaneous locomotor activity and anxiety-like behavior. Rats were placed individually in the center of a square arena (100 cm × 100 cm × 50 cm) and allowed to explore freely for 10 min. Their locomotor trajectories were recorded and analyzed using VisuTrack software (Shanghai Xinruan Information Technology Co., Ltd., Shanghai, China). The total distance traveled was calculated to evaluate locomotor activity, and the percentage of time spent in the central zone was analyzed as an index of anxiety-like behavior. The apparatus was cleaned with 75% ethanol between trials to eliminate olfactory cues.

#### 2.3.2 Y-maze test

Spatial working memory was evaluated using the Y-maze spontaneous alternation test. The maze consisted of three identical arms (labeled A, B, and C; 50 cm × 10 cm × 20 cm) diverging at 120° angles. Rats were placed at the end of one arm and allowed to explore the maze freely for 10 min. Animal movements were tracked using VisuTrack software. An alternation was defined as consecutive entries into all three arms (e.g., ABC, BCA, or CAB). The percentage of spontaneous alternation (SAR) was calculated using the formula: SAR (%) = (Number of alternations)/(Total arm entries −2) × 100.

#### 2.3.3 Rotarod test

Motor coordination and balance were assessed using a rotarod apparatus (Shanghai Xinruan Information Technology Co., Ltd., Shanghai, China). Before testing, rats were trained for 3 consecutive days to adapt to the rotating rod. On the test day, the rod accelerated from 4 to 40 rpm over a period of 5 min. The latency to fall (duration on the rod) was recorded for each rat. The average of 3 trials was used for statistical analysis.

### 2.4 Tissue sample collection and preparation

After the final experimental procedure, animals were subjected to a single terminal anesthesia using 2.5% tribromoethanol (250 mg/kg, i.p.; T48402, Sigma-Aldrich, St. Louis, MO, United States of America) prior to tissue collection. This anesthetic was used only for terminal tissue harvesting rather than for any survival procedure. Upon confirmation of deep anesthesia, as indicated by loss of pedal reflex, animals were euthanized by decapitation and the whole brain was rapidly excised on an ice-cold plate and weighed. To preserve tissue morphology, the brain tissue was flash-frozen in isopentane chilled with liquid nitrogen and subsequently stored at −80 °C prior to AFADESI-MSI analysis. For MSI experiments, we removed brain samples from −80 °C storage and equilibrated them at −20 °C for 6 h. We then sectioned the brains sagittally into 10 µm–thick slices using a cryostat (Leica, Germany). We mounted the sections directly onto positively charged microscope slides, vacuum-dried them for 15 min, and subsequently subjected them to AFADESI-MSI analysis (Cheng et al., 2025).

### 2.5 Western blot analysis

Left striatal tissue collected from the MSI/WB subset was homogenized in ice-cold RIPA lysis buffer containing protease inhibitors. After centrifugation, the supernatants were collected and protein concentrations were determined using a BCA protein assay kit. Equal amounts of protein were separated by SDS–PAGE and transferred onto PVDF membranes. After blocking with 5% non-fat milk, membranes were incubated overnight at 4 °C with primary antibodies against tyrosine hydroxylase (TH; Tyrosine Hydroxylase Recombinant Rabbit mAb, Catalog No. S0B0880, STARTER), Tom20 (Rabbit anti-TOM20 Polyclonal Antibody, Catalog No. abs146668, Absin), and the corresponding internal control. Membranes were then incubated with HRP-conjugated secondary antibodies, and immunoreactive bands were visualized using enhanced chemiluminescence. Band intensities were quantified using ImageJ software. Relative protein expression was normalized to the internal control, and Tom20 was used as the mitochondrial marker in the validation analysis. Representative blots and quantification are shown in Supplementary Figure S1.

### 2.6 AFADESI-MSI analysis conditions

We performed mass spectrometry imaging using an AFADESI-MSI platform (Chemmind Technologies, China) coupled to a Q Exactive mass spectrometer (Thermo Fisher Scientific, United States). We used a nebulization solvent consisting of acetonitrile and water (8:2, v/v) at a flow rate of 6 μL/min. We set the nebulization voltage to 3.0 kV and maintained the auxiliary gas pressure at 0.7 MPa. We acquired data in both positive and negative ion modes across a mass range of m/z 100–1,000 at a resolution of 120,000. We conducted imaging using a continuous raster-scanning mode with a scanning speed of 0.2 mm/s and a line spacing of 0.2 mm. We maintained a vertical distance of approximately 3 mm between the nebulizer needle and the tissue surface, with a nebulization angle of 60°. We set the ion transfer tube temperature to 350 °C.

### 2.7 Data processing and statistical analysis

Raw MSI data files were converted to.cdf format using Xcalibur software (Thermo Scientific) and imported into MassImager software (Chemmind Technologies, Beijing, China) for background subtraction, normalization, and ion image reconstruction. Ten anatomical regions of interest (ROIs)—olfactory bulb (OB), cortex (CTX), striatum (STR), hippocampus (HIPP), thalamus (THA), hypothalamus (HYP), pineal gland (PIN), midbrain (MB), cerebellum (CB), and pons/medulla oblongata (PONS_MY)—were manually delineated in MassImager according to the Paxinos and Watson rat brain atlas and were cross-referenced with adjacent hematoxylin-eosin (HE)-stained sections to improve anatomical accuracy (Kleven et al., 2023). For each metabolite, the mean normalized ion intensity within each ROI was calculated for each animal and used as the statistical unit for downstream analysis.

During ROI selection, the average mass spectral peak intensity for each region was extracted. The AFADESI-MSI method employed in this study achieved a spatial resolution of 100 μm, sufficient to resolve microregional metabolic differences, and demonstrated high sensitivity for small-molecule metabolites across both positive and negative ion modes (*m/z* 100–1,000), enabling detection of metabolites at physiologically relevant concentrations.

Multivariate analyses were performed in R (version 4.3.2). Principal component analysis (PCA) and orthogonal partial least squares discriminant analysis (OPLS-DA) were conducted using the ropls package to evaluate differences among the Control, MPTP, and Nicotine groups. OPLS-DA model robustness was assessed by 5-fold cross-validation and 200 permutation tests (Thévenot et al., 2015). Differential metabolites were screened using a combined criterion of Variable Importance in Projection (VIP) > 1.0 and univariate P < 0.05. For univariate analysis, data distribution was first assessed for normality. Student’s t-test was applied to normally distributed data, whereas the Mann–Whitney U test was used when normality assumptions were not met. For targeted quantitative validation data and behavioral outcomes involving three groups, one-way analysis of variance (ANOVA) followed by Tukey’s *post hoc* test was used. We identified metabolites by accurate mass-to-charge ratios (mass error <5 ppm) through database matching against Human Metabolome Database (HMDB) (http://www.hmdb.ca) and METLIN (http://metlin.scripps.edu/). Representative high-resolution spectra of selected ions are provided in Supplementary Figure S3. Pathway enrichment analysis was performed using the pathway analysis module of MetaboAnalyst 6.0, which combines over-representation analysis with pathway topology analysis (Pang et al., 2024). Pathways meeting the predefined significance criteria (raw P < 0.05 and pathway impact >0.10) were considered enriched. Semi-quantitative bar plots shown alongside MSI ion images were generated from ROI-averaged normalized intensities for each animal.

## 3 Results

### 3.1 Whole-brain spatial metabolic profiling overview

**FIGURE 1:**
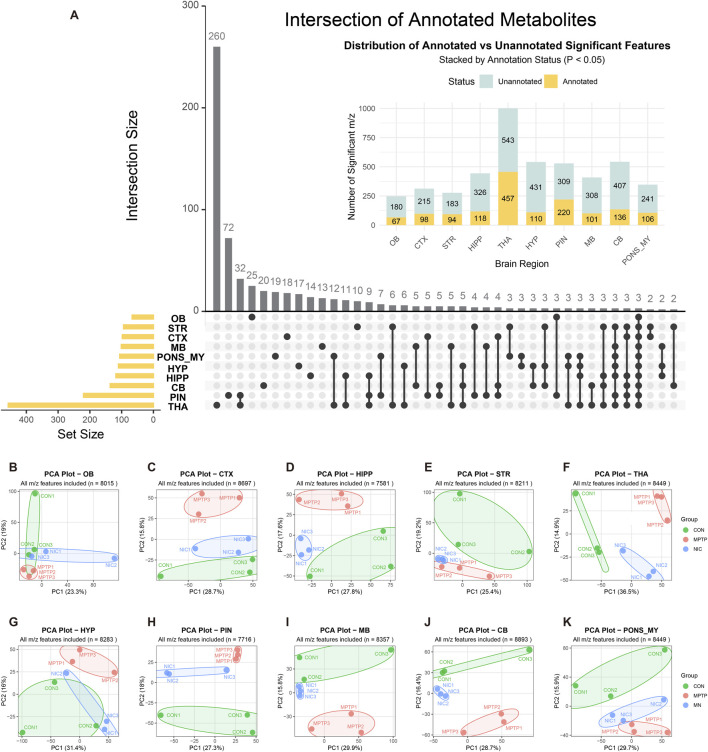
Spatial metabolomics reveal MPTP-induced metabolic reprogramming and systematic reversal effects of nicotine. **(A)** UpSet plot showing quantitative distribution of significantly different metabolomic features *(P < 0.05*) across the ten anatomical brain regions and their intersections. Vertical bar charts represent the number of features within specific intersections, while horizontal bar charts indicate the total number of differential features in each brain region. Stacked colors distinguish annotated (yellow) and unannotated (blue) metabolites. **(B–K)** Principal component analysis (PCA) plots constructed based on full-spectrum metabolomic features of the ten different brain regions: olfactory bulb (OB), cortex (CTX), hippocampus (HIPP), striatum (STR), thalamus (THA), hypothalamus (HYP), pineal gland (PIN), midbrain (MB), cerebellum (CB), and pons/medulla oblongata (PONS_MY). Cluster colors represent different groups: control group (CON, green), MPTP model group (MPTP, red), and nicotine treatment group (NIC, blue). Elliptical regions represent 95% confidence intervals, showing clustering and separation trends of metabolic profiles among groups.

To systematically characterize metabolic reprogramming during MPTP-induced PD pathogenesis and to evaluate the modulatory effects of nicotine, we performed untargeted spatial metabolomic analysis of whole-brain rat sections using AFADESI-MSI. As shown in Figure 1A, we detected a large number of metabolomic features with statistically significant differences (*P < 0.05*) across ten anatomical brain microregions. Among these regions, the THA showed the strongest metabolic response, with more than 1,000 significantly altered features, including 457 annotated metabolites, ranking first among all brain regions. The HYP and PIN followed, with 110 and 220 differential metabolites identified, respectively. The UpSet plot shown in the bottom left of Figure 1A further demonstrated pronounced spatial heterogeneity in metabolic alterations. Although several metabolic features were shared across multiple brain regions, the THA exhibited exceptionally high regional specificity, with 260 unique differential features (Intersection Size), far exceeding those observed in other regions. Notably, the PIN, despite its small anatomical size and endocrine function, also displayed 72 region-specific differential features. These findings strongly indicate that, beyond the classical nigrostriatal pathway, the thalamus and pineal gland may play critical and previously underappreciated roles in the pathological progression of PD. Behavioral and orthogonal validation data are summarized in Supplementary Figure S1. In brief, nicotine improved MPTP-induced deficits in locomotor activity, spatial working memory, and motor coordination, and these phenotypic changes were accompanied by independent striatal LC-MS and WB measurements consistent with the MSI-based observations.

To visualize global metabolic differences among the experimental groups, we conducted PCA separately for each of the ten brain regions (Figures 1B–K). In all analyzed regions, particularly the STR (Figure 1E) and MB (Figure 1I), the PCA score plots showed a clear separation between the MPTP model group and the control group (CON), confirming pronounced metabolic trajectory shifts in the PD model. Notably, samples from the nicotine treatment group (NIC) consistently clustered between the CON and MPTP groups or exhibited a clear shift toward the CON group. These patterns indicate that nicotine does not act on a single metabolic target but broadly mitigates MPTP-induced metabolic disturbances across multiple brain regions, supporting its systemic neuroprotective effect.

### 3.2 Screening and identification of differential metabolites in key brain regions

Although the whole-brain analysis revealed widespread metabolic alterations, we performed a focused regional analysis to more precisely define the neuroprotective mechanism of nicotine. We selected six key brain regions for detailed investigation based on two criteria. First, pathological relevance: the STR and MB are central to dopaminergic neuron degeneration and motor symptom development in PD; the HIPP plays a critical role in cognition and memory; and the PIN and HYP are major regulatory centers for circadian rhythms and autonomic nervous function. Second, metabolic activity: based on whole-brain feature counts (Figure 1A), the THA, HYP, and PIN showed the highest numbers of differential metabolic features and the strongest regional specificity, indicating pronounced metabolic activity during MPTP-induced reprogramming.

**FIGURE 2:**
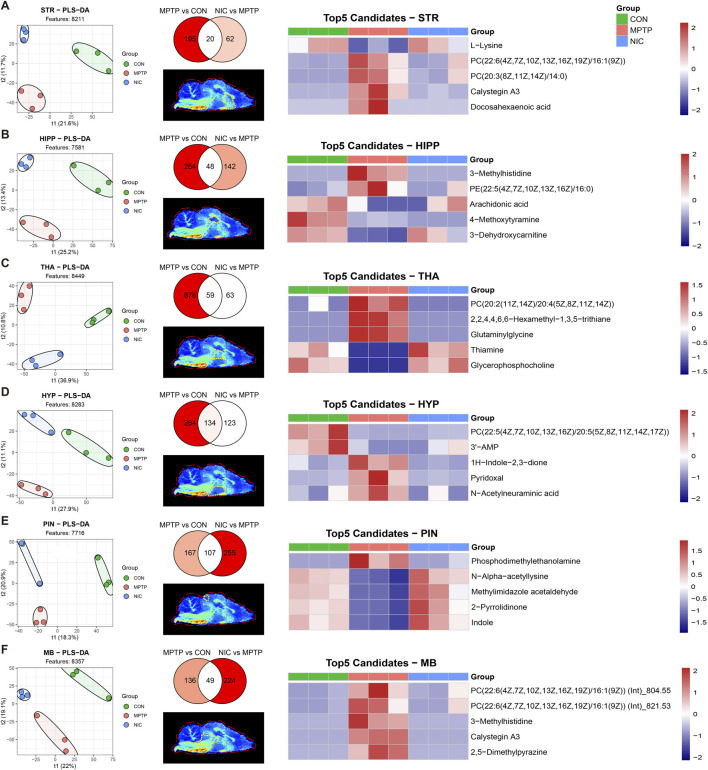
Multidimensional screening and visualization of region-specific differential metabolites in key brain regions. **(A–F)** Differential metaboliges in six key functional brain regions: STR, HIPP, THA, HYP, PIN, and MB. Left: PLS-DA score plots showing distinct separation of metabolic phenotypes among the CON, MPTP, and NIC groups. Middle: Venn diagrams illustrating numbers of overlapping metabolites between the “MPTP vs. CON” model-induced differential group and the “NIC vs. MPTP” drug-administered differential group. Overlapping metabolites are defined as nicotine-responsive metabolites. Insets are schematic diagrams of the anatomical localization of corresponding brain regions in MSI.

For these six ROIs, we first constructed partial least squares discriminant analysis (PLS-DA) models (Figures 2A–F, left panels). The score plots showed clear separation among the control (CON), MPTP, and nicotine-treated (NIC) groups in each region, demonstrating that individual brain regions exhibit distinct metabolic phenotypes in response to PD pathology and nicotine intervention.

To identify the specific metabolic targets through which nicotine exerts therapeutic effects, we applied a Venn diagram–based screening strategy to define “intersecting metabolic features” that were significantly altered by MPTP (MPTP vs. CON) and effectively reversed by nicotine (NIC vs. MPTP) (Figures 2A–F, middle panels). This analysis revealed marked regional differences in the number of metabolites that responded to nicotine treatment across various brain regions.

High-response regions: The HYP and PIN showed the largest numbers of shared differential metabolites, with intersection counts of 134 and 107, respectively. These results further confirm the high sensitivity of these non–motor symptom–related regions to nicotine treatment.

Core pathological regions: The THA, MB, and HIPP contained 59, 49, and 48 intersecting metabolites, respectively.

STR: We identified 20 key intersecting metabolites in the STR.

Based on Variable Importance in Projection scores (VIP >1), we further selected the top five candidate biomarkers for each brain region. We visualized group-wise abundance changes using heatmaps (Figures 2A–F, right panels). These biomarkers primarily involve lipid metabolism, amino acid metabolism, and energy-related pathways.

STR: The top five metabolites include L-lysine, two phosphatidylcholines(PC) species, (PC (22:6/16:1) and PC (20:3/14:0)), the alkaloid calystegin A3, and the neuroprotective fatty acid docosahexaenoic acid (DHA).

HIPP: Key metabolites include 3-methylhistidine, a phosphatidylethanolamine(PE) species, (PE(22:5/16:0)), arachidonic acid (a precursor of inflammatory mediators), 4-methoxytyramine, and 3-dehydroxycarnitine.

THA: The most significantly altered metabolites include PC(20:2/20:4), 2,2,4,4,6,6-hexamethyl-1,3,5-trithiane, glutaminylglycine, thiamine (an essential coenzyme in energy metabolism), and glycerophosphocholine (a membrane component).

HYP: Highly responsive metabolites include PC (22:5/20:5), the energy-related molecule 3′-AMP, the indole derivative 1H-indole-2,3-dione, pyridoxal (a form of vitamin B6), and N-acetylneuraminic acid.

PIN: Region-specific biomarkers mainly include phosphodimethylethanolamine, N-alpha-acetyllysine, the histidine-derived metabolite methylimidazole acetaldehyde, 2-pyrrolidinone, and indole.

MB: The top five metabolites consist of two different adduct forms of PC (22:6/16:1), 3-methylhistidine, calystegin A3, and 2,5-dimethylpyrazine.

These heatmaps confirm the potential of these small molecules as PD biomarkers and clearly illustrate nicotine’s broad regulatory effects on membrane lipid repair (PC/PE), anti-inflammatory pathways (DHA/arachidonic acid), and neurotransmitter precursor metabolism.

### 3.3 *In situ* drug distribution and neurotransmitter network remodeling

**FIGURE 3:**
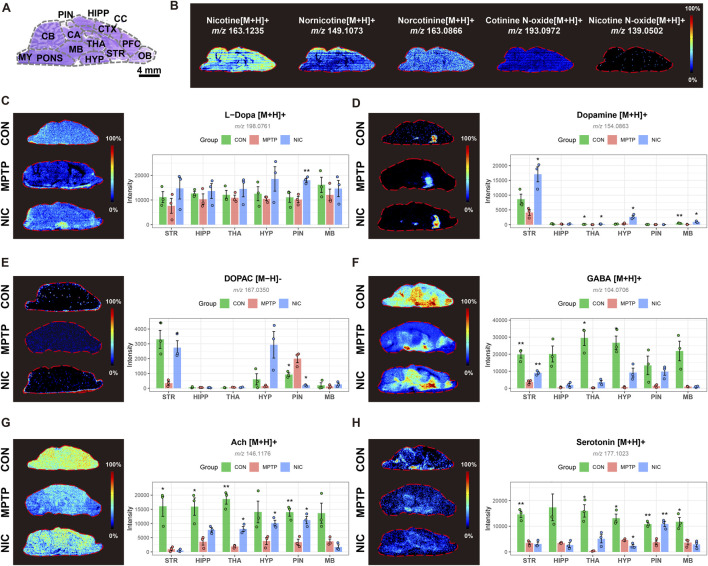
*In situ* Visualization of Nicotine and its Metabolites and Spatial Remodeling of the Neurotransmitter Network. **(A)** Schematic diagram of a sagittal section of the rat brain with annotations of each anatomical microregion. **(B)** Representative MSI ion images showing spatial distribution of nicotine and its major *in vivo* metabolites (Nornicotine, Norcotinine, Cotinine N-oxide, and Nicotine N-oxide) in brain tissue, confirming brain penetration ability of the drug. **(C–H)** MSI ion images (left) and semi-quantitative bar charts (right) of key neurotransmitters and their metabolites, including L-DOPA, Dopamine, DOPAC, GABA, Acetylcholine (ACh), and Serotonin. Ion images are displayed as normalized intensity heat maps, where red indicates relatively high signal intensity and blue indicates relatively low signal intensity. The same color scale was applied across groups for each metabolite, whereas color scales were not compared across different metabolites. Quantitative bar plots were generated from ROI-averaged normalized intensities for each animal and are presented as mean ± standard error of the mean (SEM). Compared with the MPTP group, *P < 0.05, **P < 0.01, ***P < 0.001.

Clarifying the spatial distribution of drugs within the brain is essential for understanding their pharmacological mechanisms. Leveraging the label-free imaging capability of AFADESI-MSI, we first assessed whether nicotine can cross the blood–brain barrier (BBB). As shown in Figure 3B, we detected clear ion signals corresponding to nicotine and its major metabolites, including nornicotine and cotinine N-oxide, throughout the brain parenchyma. These signals displayed broad and heterogeneous distribution across multiple brain regions, demonstrating that nicotine efficiently penetrates the BBB and reaches diverse intracerebral targets.

Based on these findings, we next examined the regulatory effects of nicotine on the brain neurotransmitter network. In the STR and MB, which are directly linked to PD motor symptoms, MPTP administration caused marked depletion of dopamine (DA) (Figure 3D), its biosynthetic precursor L-DOPA (Figure 3C), and its primary metabolite 3,4-dihydroxyphenylacetic acid (DOPAC) (Figure 3E). These changes closely reflect the degeneration and loss of dopaminergic neurons characteristic of PD pathology. Nicotine treatment significantly restored the levels of these dopaminergic markers (*P < 0.05*), indicating that nicotine either protects dopaminergic nerve terminals or enhances DA synthesis and release.

Spatial metabolomic analysis further revealed that nicotine exerts broad regulatory effects beyond the dopaminergic system. In brain regions associated with cognition and emotion, including the HIPP and THA, MPTP significantly reduced levels of the inhibitory neurotransmitter γ-aminobutyric acid (GABA) (Figure 3F) and the mood-regulating neurotransmitter serotonin (Figure 3H). Nicotine treatment effectively reversed these deficits, restoring GABA and serotonin to near-control levels. These results indicate that nicotine not only rescues dopaminergic dysfunction but also rebalances inhibitory and mood-related neurotransmitter networks, providing a metabolic basis for its beneficial effects on both motor and non-motor symptoms of PD.

### 3.4 Restoration of mitochondrial energy metabolism and alleviation of oxidative stress

**FIGURE 4:**
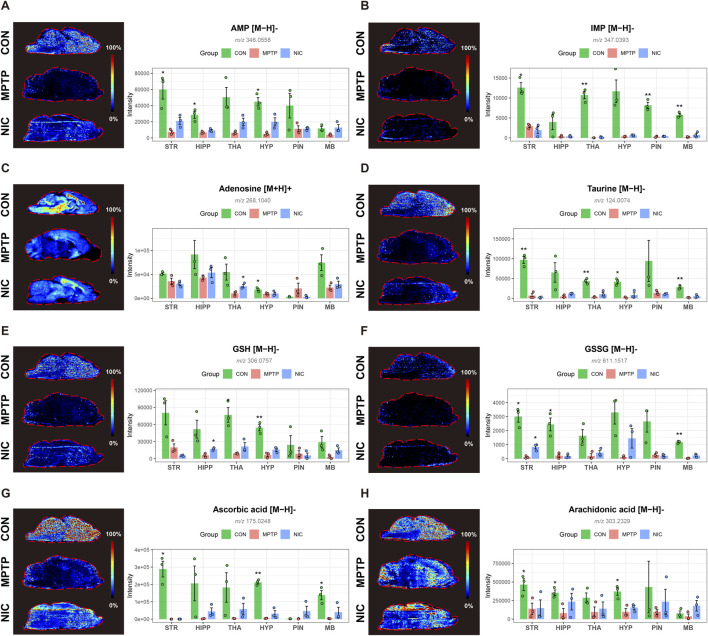
Nicotine Ameliorates MPTP-Induced Mitochondrial Energy Failure and Oxidative Stress. **(A–C)** Spatial distribution and quantitative analysis of energy metabolism-related biomarkers: AMP, IMP, and adenosine. Abnormal elevation of AMP indicates energy depletion, which was reversed by nicotine treatment. **(D–G)** MSI visualization and quantification of oxidative stress-related metabolites, including taurine, reduced GSH, GSSG, and ascorbic acid. Nicotine significantly restores levels of GSH and ascorbic acid. **(H)** Spatial distribution of arachidonic acid, a precursor of inflammatory mediators. Red dashed lines outline the whole-brain contour. Quantitative bar plots were generated from ROI-averaged normalized intensities for each animal and are presented as means ± SEM. Compared with the MPTP group, *P < 0.05, **P < 0.01, ***P < 0.001.

Mitochondrial dysfunction and oxidative stress represent central mechanisms underlying MPTP-induced neurotoxicity. Spatial metabolomic imaging captured marked alterations in energy- and redox-related metabolites and their partial normalization following nicotine treatment. At the level of energy metabolism, rats in the MPTP group showed marked metabolic abnormalities in adenosine monophosphate (AMP) (Figure 4A) and inosine monophosphate (IMP; Figure 4B) in the STR and THA, accompanied by altered adenosine levels (Figure 4C). These changes are consistent with disturbed purine turnover and energy stress in vulnerable regions. Nicotine treatment significantly attenuated these abnormalities, suggesting partial recovery of mitochondria-associated metabolic status rather than direct proof of restored respiratory chain function. In parallel, taurine levels (Figure 4D) declined markedly in the striatum and midbrain after MPTP exposure, reflecting reduced metabolic stress-buffering capacity.

MPTP also disrupted the brain antioxidant system. Signals for reduced glutathione (GSH) (Figure 4E) and ascorbic acid (Figure 4G) decreased significantly in the STR and MB regions, whereas oxidized glutathione (GSSG; Figure 4F) showed abnormal redistribution, together indicating impaired redox homeostasis. Nicotine treatment significantly reversed these changes. Importantly, these MSI-based findings were directionally supported by independent striatal LC-MS measurements of dopamine-related and glutathione-related metabolites, as well as WB analysis of TH and Tom20 in Supplementary Figure S1. In addition, supplementary targeted LC-MS measurements of pathway-related metabolites further supported nicotine-associated normalization of redox- and lipid-related metabolic disturbances (Supplementary Figure S2). Furthermore, arachidonic acid (Figure 4H), a precursor of major inflammatory mediators, exhibited marked disturbance in the MPTP group, and nicotine partially corrected this dysregulation.

### 3.5 Brain region-specific metabolic pathway enrichment analysis

**FIGURE 5:**
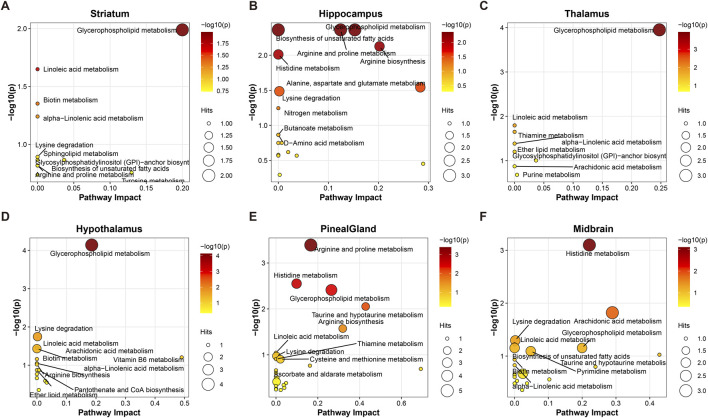
KEGG pathway enrichment analysis reveals region-specific mechanisms of nicotine action. Bubble charts of pathway enrichment analysis for differential metabolites in the STR **(A)**, HIPP **(B)**, THA **(C)**, HYP **(D)**, PIN **(E)**, and MB **(F)**, respectively. The X-axis represents the Pathway Impact value (reflecting topological importance of affected metabolites within the pathway), and the Y-axis represents enrichment significance (-log_10_(P)). The size of each bubble is proportional to the number of differential metabolites mapped to the corresponding pathway. The color gradient (from yellow to red) indicates an increasing level of significance. Glycerophospholipid metabolism is identified as a key common pathway across brain regions.

To clarify the biological functions associated with the identified differential metabolites, we mapped these metabolites to the KEGG database and performed pathway enrichment analysis for the six ROIs (Figure 5). Glycerophospholipid metabolism emerged as a recurrent enriched pathway (*P < 0.05*, Impact >0.1) across several key brain regions, including the striatum (Figure 5A), hippocampus (Figure 5B), and thalamus (Figure 5C). This finding is consistent with the widespread alterations in phosphatidylcholine (PC) and phosphatidylethanolamine (PE) species observed in the mass spectrometry imaging(MSI) data and supports glycerophospholipid metabolism as a statistically recurrent pathway associated with nicotine-related metabolic remodeling rather than as a standalone definitive mechanism.

In addition to these shared pathways, we identified region-specific metabolic signatures. Histidine metabolism showed prominent enrichment in the pineal gland (Figure 5E) and midbrain (Figure 5F), whereas linoleic acid metabolism was more apparent in the striatum and thalamus. Together, these results suggest that nicotine is associated with both common and region-selective metabolic responses across the PD brain.

### 3.6 Validation of specific enriched pathways and visualization of representative metabolites

**FIGURE 6:**
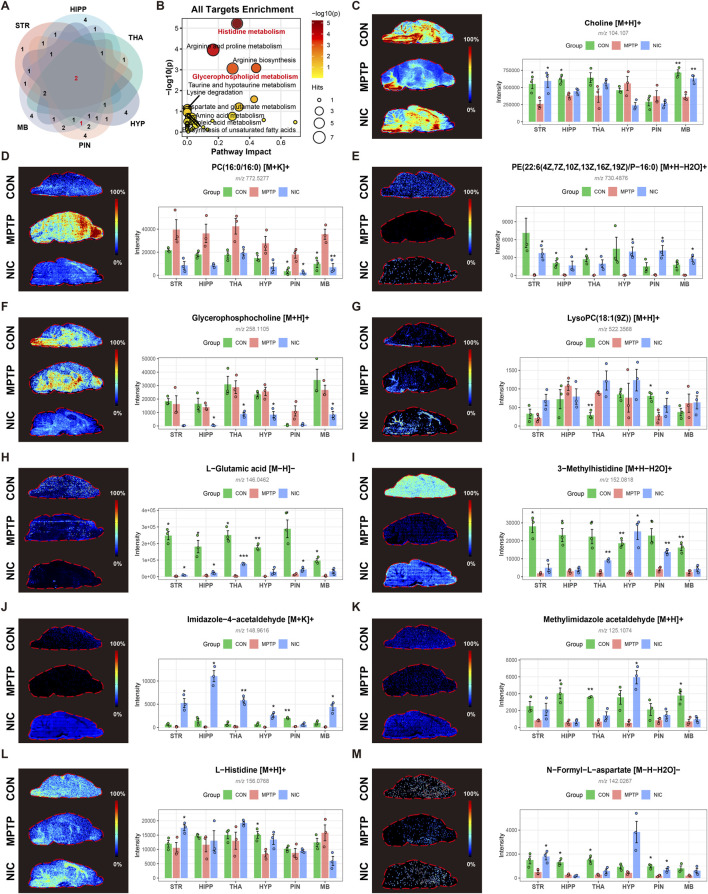
In-Depth Analysis of Key Metabolic Pathways (Glycerophospholipid Metabolism and Histidine Metabolism) and their Core Metabolites. **(A)** Venn diagram showing intersection of significantly enriched pathways among the six brain regions. Red numbers highlight the pervasiveness of glycerophospholipid metabolism as a shared pathway. **(B)** Comprehensive overview of pathway impact values based on whole-brain region data, emphasizing the high significance of histidine and glycerophospholipid metabolism. **(C–G)** MSI imaging and quantification of key metabolites related to the glycerophospholipid metabolic pathway: Choline, PC(16:0/16:0), PE(22:6/16:0), Glycerophosphocholine, and LysoPC(18:1). Restoration of these lipid molecules suggests restoration of cell membrane integrity. **(H–M)** MSI imaging and quantification of metabolites related to the histidine metabolic pathway: L-Glutamic acid, 3-Methylhistidine, Imidazole-4-acetaldehyde, Methylimidazole acetaldehyde, L-Histidine, and N-Formyl-L-aspartate. Special attention is paid to significant changes in these metabolites in the PIN. Quantitative bar plots were generated from ROI-averaged normalized intensities for each animal and are presented as means ± SEM. Compared with the MPTP group, *P < 0.05, **P < 0.01, ***P < 0.001.

To further examine the pathway enrichment results, we focused on glycerophospholipid metabolism, which showed region-preferential enrichment (Figure 6). The Venn diagram in Figure 6A illustrates the overlap of metabolites assigned to glycerophospholipid metabolism among different brain regions. To summarize pathway-level trends, we integrated enrichment results across regions and generated a pan-target enrichment plot (Figure 6B). In this integrated analysis, glycerophospholipid metabolism showed both high significance and high pathway impact, supporting it as a prominent shared pathway associated with nicotine-responsive metabolic remodeling.

Within the glycerophospholipid metabolism pathway, MSI revealed marked decreases in choline (Figure 6C), phosphatidylcholine PC(16:0/16:0) (Figure 6D), and glycerophosphocholine (Figure 6F) in the MPTP group, particularly in the STR and MB. Nicotine treatment partially restored these membrane lipid-related metabolites. Consistent with these MSI observations, supplementary targeted LC-MS data further supported nicotine-associated normalization of multiple lipid-related metabolites, including choline and lysophospholipid-related species (Supplementary Figure S2). Together with the pathway map provided in Supplementary Figures S4,3, these findings support disruption of membrane lipid homeostasis as an important component of the observed metabolic phenotype. Consistently, the altered distribution of a lysophosphatidylcholine species, LysoPC(18:1(9Z)) (Figure 6G) further confirmed that nicotine reestablishes membrane lipid homeostasis in PD-affected brain regions.

For the histidine metabolism pathway, MSI revealed widespread perturbation of related metabolites across several anatomical regions. L-glutamic acid (Figure 6H), a metabolite functionally linked to excitatory amino acid metabolism, exhibited pronounced accumulation in the STR and CTX of the MPTP group. Nicotine treatment reduced these elevated signals, suggesting attenuation of excitatory metabolic stress. Downstream metabolites within the histidine metabolism pathway also showed marked alterations in regions including the HIPP, STR, and PIN. Specifically, 3-methylhistidine (Figure 6I) and imidazole-4-acetaldehyde (Figure 6J) were significantly elevated in these regions in the MPTP group, reflecting severe metabolic stress and tissue injury. In contrast, the essential amino acid L-histidine (Figure 6L) was broadly downregulated throughout the brain parenchyma, with particularly pronounced reductions in the HIPP and THA. This decrease likely reflects excessive precursor consumption under inflammatory and stress-related conditions. Notably, nicotine treatment effectively restored L-histidine levels as well as its metabolic intermediate N-formyl-L-aspartate (Figure 6M), suggesting that nicotine modulates histidine metabolism and may indirectly influence circadian and metabolic rhythms, thereby contributing to the improvement of non-motor symptoms in PD.

## 4 Discussion

Parkinson’s disease is increasingly recognized as a disorder of distributed brain network dysfunction rather than a lesion confined to the nigrostriatal pathway (Wheeler et al., 2024; Yang et al., 2020; Cheng et al., 2025; Paul et al., 2023). In this context, the major contribution of the present study is not merely the identification of altered metabolites, but the demonstration that nicotine reshapes the PD metabolic landscape in a spatially heterogeneous manner across anatomically and functionally distinct brain regions (Wheeler et al., 2024; Neumann, 2025; Vicari et al., 2024). By combining whole-brain AFADESI-MSI with ROI-based analysis, we show that nicotine-associated metabolic remodeling is better understood as a coordinated multi-region response involving neurotransmitter remodeling, restoration of mitochondrial and redox homeostasis, and recovery of membrane lipid balance, rather than as a single-target effect.

An important aspect of this study is the rationale for examining ten anatomical brain regions (Farzana et al., 2023). These regions were selected to cover complementary dimensions of PD-related vulnerability and drug response: the striatum and midbrain represent the core dopaminergic circuitry underlying classical motor impairment; the cortex and hippocampus are closely linked to cognition and affective regulation; the thalamus and hypothalamus act as major relay and homeostatic centers; the pineal gland provides a neuroendocrine and circadian node; and the olfactory bulb, cerebellum, and pons/medulla oblongata extend the analysis to additional regions relevant to early PD involvement, motor coordination, and autonomic/brainstem function. This design allowed us to move beyond a restricted lesion-centered view and instead evaluate whether metabolic remodeling follows a broader spatial logic across the brain (Yang et al., 2020; Cheng et al., 2025). Notably, the thalamus and pineal gland exhibited particularly strong metabolic responses and marked region-specificity, suggesting that these structures may represent underappreciated components of PD-related metabolic dysregulation.

The region-specific neurotransmitter findings further support this network-level interpretation (Shariatgorji et al., 2019; Fridjonsdottir et al., 2021; Zheng et al., 2025). In the striatum and midbrain, nicotine restored dopamine-related signals, which is consistent with the central role of these regions in dopaminergic degeneration and motor dysfunction. However, the significance of the present findings extends beyond dopamine. The normalization of GABA and serotonin in the hippocampus and thalamus suggests that nicotine may also influence non-motor symptom-related circuitry, including pathways linked to anxiety, emotional processing, and cognitive dysfunction (Yang et al., 2020; Fridjonsdottir et al., 2021; Zheng et al., 2025). This point is important because non-motor symptoms in PD are often insufficiently explained by dopamine depletion alone. Our data therefore support the view that nicotine does not simply compensate for neurotransmitter loss in the nigrostriatal pathway, but may act by rebalancing multiple neurotransmitter networks across functionally distinct microregions.

A second major implication of the study is that metabolic rescue by nicotine appears to converge on mitochondrial function and redox stability. The abnormal accumulation of AMP/IMP together with altered adenosine strongly suggests impaired ATP turnover and energetic stress in the MPTP brain, especially in the striatum and thalamus. Likewise, the depletion of GSH and ascorbic acid, accompanied by disturbance of GSSG, indicates a pronounced oxidative imbalance. Nicotine partially reversed these changes, supporting the interpretation that its protective effect involves improved metabolic resilience rather than neurotransmitter regulation alone. In PD, mitochondrial dysfunction, oxidative stress, and neurotransmitter failure are tightly interconnected. The present spatial data suggest that nicotine may intervene at this intersection by attenuating local energy crisis and restoring antioxidant buffering capacity in vulnerable regions. Our pathway analysis further indicates that glycerophospholipid metabolism may represent a shared mechanistic backbone of nicotine action across multiple brain regions (Jha et al., 2024; Yang et al., 2020; Li et al., 2025). This observation is biologically meaningful because membrane phospholipids are not only structural components of neuronal and mitochondrial membranes, but also directly influence receptor localization, synaptic vesicle dynamics, lipid signaling, and susceptibility to oxidative injury. Thus, the repeated involvement of phosphatidylcholine- and phosphatidylethanolamine-related metabolites across regions suggests that nicotine may stabilize membrane homeostasis at a systems level. From this perspective, restoration of membrane lipid balance may provide a common biochemical foundation upon which more region-specific effects—such as dopaminergic remodeling in the striatum/midbrain or non-dopaminergic modulation in the hippocampus/thalamus—are superimposed.

Another noteworthy finding is the strong metabolic responsiveness of the pineal gland and the enrichment of histidine-related metabolites in this region. Although this observation remains exploratory, it raises the possibility that nicotine may influence PD-related circadian or neuroendocrine dysregulation. Sleep disturbance and circadian abnormalities are increasingly recognized as integral components of PD, yet they are rarely incorporated into spatial metabolomic analyses. Our results do not establish a direct causal link between pineal histidine metabolism and circadian- or neuroendocrine-related processes, but they identify this axis as a plausible hypothesis-generating direction for future work. Similarly, the prominent thalamic response suggests that relay nuclei involved in sensorimotor and limbic integration may be metabolically more engaged in PD progression and nicotine response than is typically appreciated.

The methodological implications of this work also deserve emphasis (Wheeler et al., 2024). A key strength of AFADESI-MSI is that it preserves tissue architecture while enabling untargeted metabolic mapping across large brain sections, thereby avoiding the averaging effect inherent to homogenate-based metabolomics (Neumann, 2025; Vicari et al., 2024). In the present study, ROI assignment was guided by the Paxinos and Watson rat brain atlas and cross-referenced with adjacent HE-stained sections, which increased the anatomical reliability of regional segmentation (Farzana et al., 2023). This atlas-guided, morphology-assisted strategy is particularly important when comparing multiple neighboring brain structures within the same sagittal section. At the same time, the present findings should be interpreted in light of the spatial scale of the technique. The approximately 100 μm spatial resolution is sufficient for region-level and subregional metabolic comparison, but it is not adequate for cell-type-specific attribution or for resolving very fine structural boundaries (Wheeler et al., 2024; Neumann, 2025). Accordingly, our conclusions are best understood at the level of brain microregions rather than single cells.

Sensitivity is another issue that warrants explicit discussion. The dual-polarity AFADESI-MSI workflow and broad m/z acquisition window enabled detection of a wide range of small metabolites and lipids directly from tissue sections, which is a clear advantage for exploratory whole-brain metabolic profiling. However, MSI signal intensity remains semi-quantitative, and sensitivity is not uniform across metabolite classes (Wheeler et al., 2024). Low-abundance compounds, structurally similar isomers, and metabolites with poor ionization efficiency may be underrepresented (Jha et al., 2024). In addition, metabolite annotation in this study was primarily based on accurate mass matching, which provides biologically useful putative identifications but does not by itself establish full structural certainty. Therefore, the present study is most powerful for defining spatial metabolic signatures and hypothesis-generating pathways, whereas absolute quantification and definitive structural confirmation will require further targeted LC-MS/MS or MS/MS-based validation.

Although the MPTP paradigm is a widely used and well-validated toxin-based model of PD, it reproduces selected aspects of dopaminergic neurodegeneration rather than the full spectrum of human disease (Meredith and Rademacher, 2011; Smeyne and Jackson-Lewis, 2005; Jackson-Lewis and Przedborski, 2007; Klemann et al., 2016). These methodological boundaries also explain why the present discussion should not overstate causality. The data strongly support an association between nicotine treatment and region-specific metabolic restoration, but they do not prove that each altered metabolite is a direct mediator of neuroprotection. Rather, the results support a model in which nicotine acts on multiple interacting levels: it reshapes neurotransmitter balance in canonical and non-canonical PD-related regions, improves local mitochondrial/redox status, and restores membrane lipid homeostasis across the brain. Future studies integrating higher-resolution spatial approaches, targeted metabolite validation, and cell-type-specific functional experiments will be needed to define how these layers interact mechanistically (Neumann, 2025; Vicari et al., 2024; Clarke et al., 2025).

In summary, this study extends the understanding of nicotine in PD from a conventional dopamine-centered framework to a spatially resolved model of distributed metabolic rescue. The findings suggest that nicotine-associated metabolic remodeling is built upon both common metabolic programs shared across brain regions and region-specific pathway biases linked to local vulnerability and function. More broadly, our work highlights the value of spatial metabolomics for revealing how neurodegenerative pathology and drug responses are organized across the brain, and it provides a framework for interpreting PD as a disorder of regionally heterogeneous but mechanistically interconnected metabolic failure.

## 5 Conclusion

In summary, this study achieved high-resolution visualization of metabolic profiles across brain microregions using AFADESI-MSI. Comprehensive spatial metabolomic analysis provided detailed insight into both systemic and region-specific metabolic reprogramming induced by MPTP in PD. Nicotine markedly alleviated these metabolic disturbances by rebalancing striatal dopaminergic signaling, restoring mitochondrial energy metabolism, repairing global membrane lipid homeostasis, and modulating pineal gland–specific metabolic pathways. These findings support the value of spatial metabolomics for investigating brain-region-specific metabolic remodeling in PD and provide spatially resolved evidence that nicotine is associated with multi-region metabolic responses in this experimental model.

## Data Availability

The original contributions presented in the study are included in the article/Supplementary Material, further inquiries can be directed to the corresponding authors.
