# Supplementary figures and images for: Exploring In situ neuroprotective mechanisms of nicotine in an MPTP-Induced Parkinson’s disease rat model using spatial metabolomics
Source: Front Cell Dev Biol. 2026 May 1;14:1818201. doi: 10.3389/fcell.2026.1818201 (PMC13175961; doi:10.3389/fcell.2026.1818201)

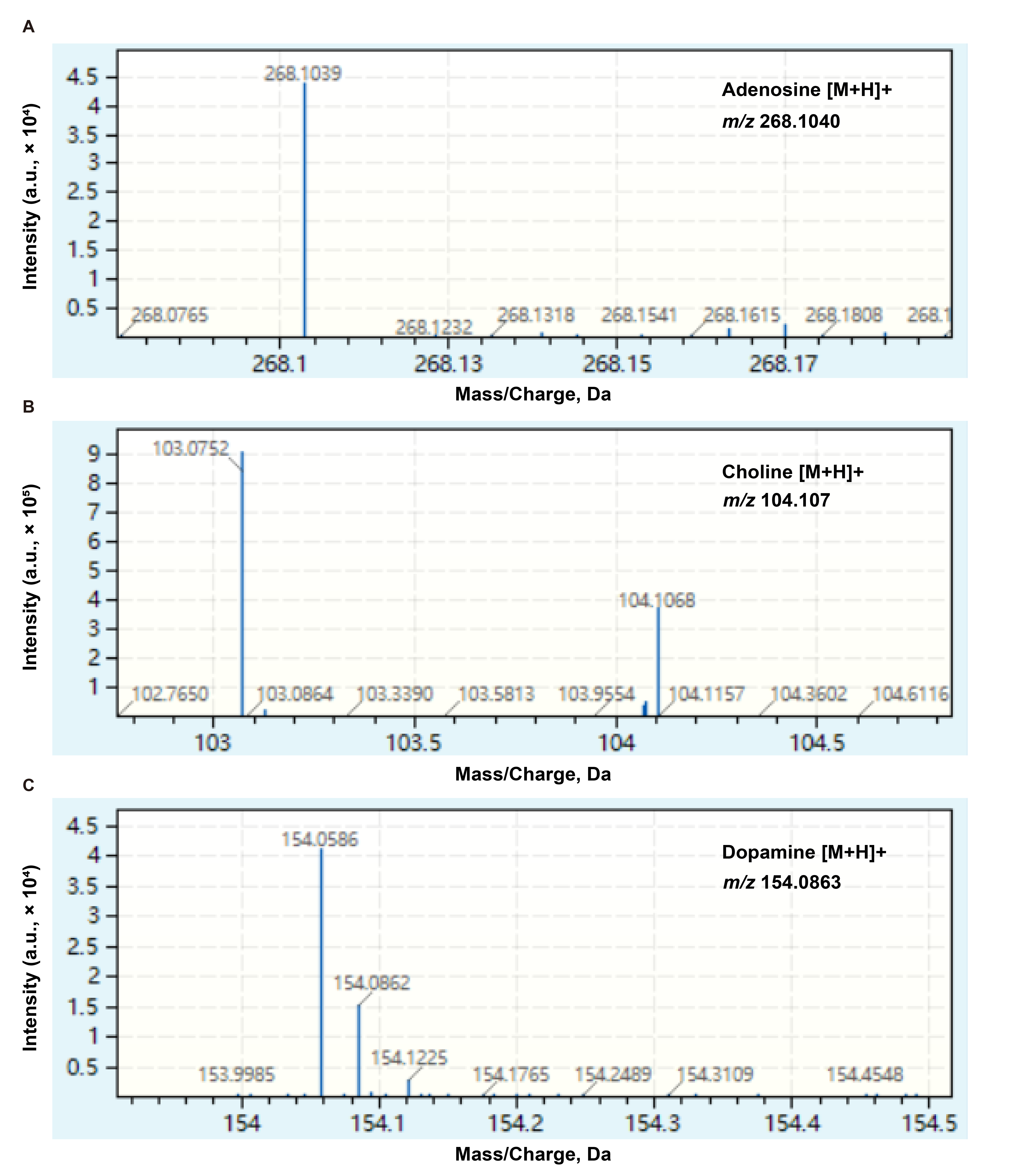

Supplement: Supplementary file 1 [file Image3.tif]

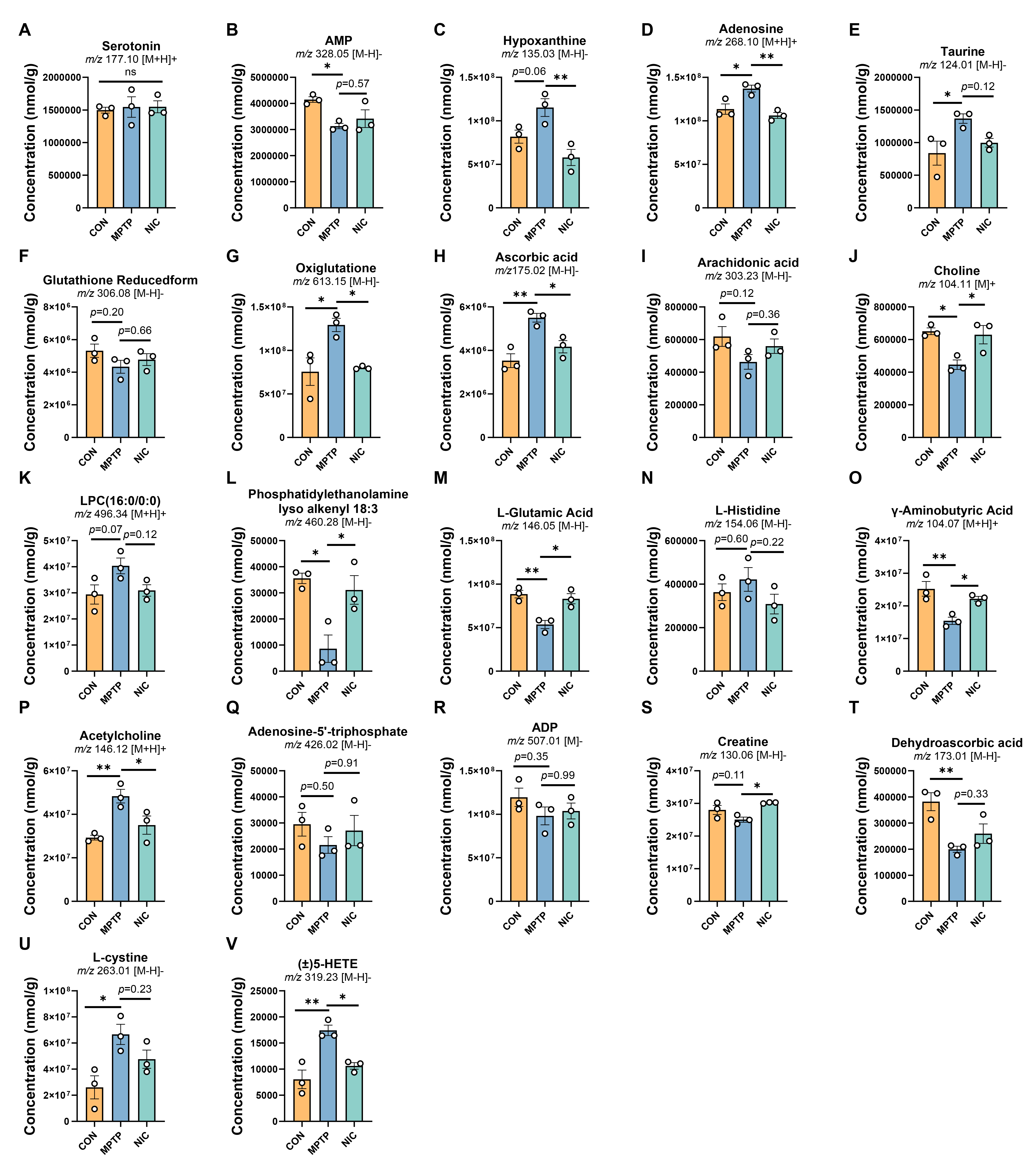

Supplement: Supplementary file 2 [file Image2.tif]

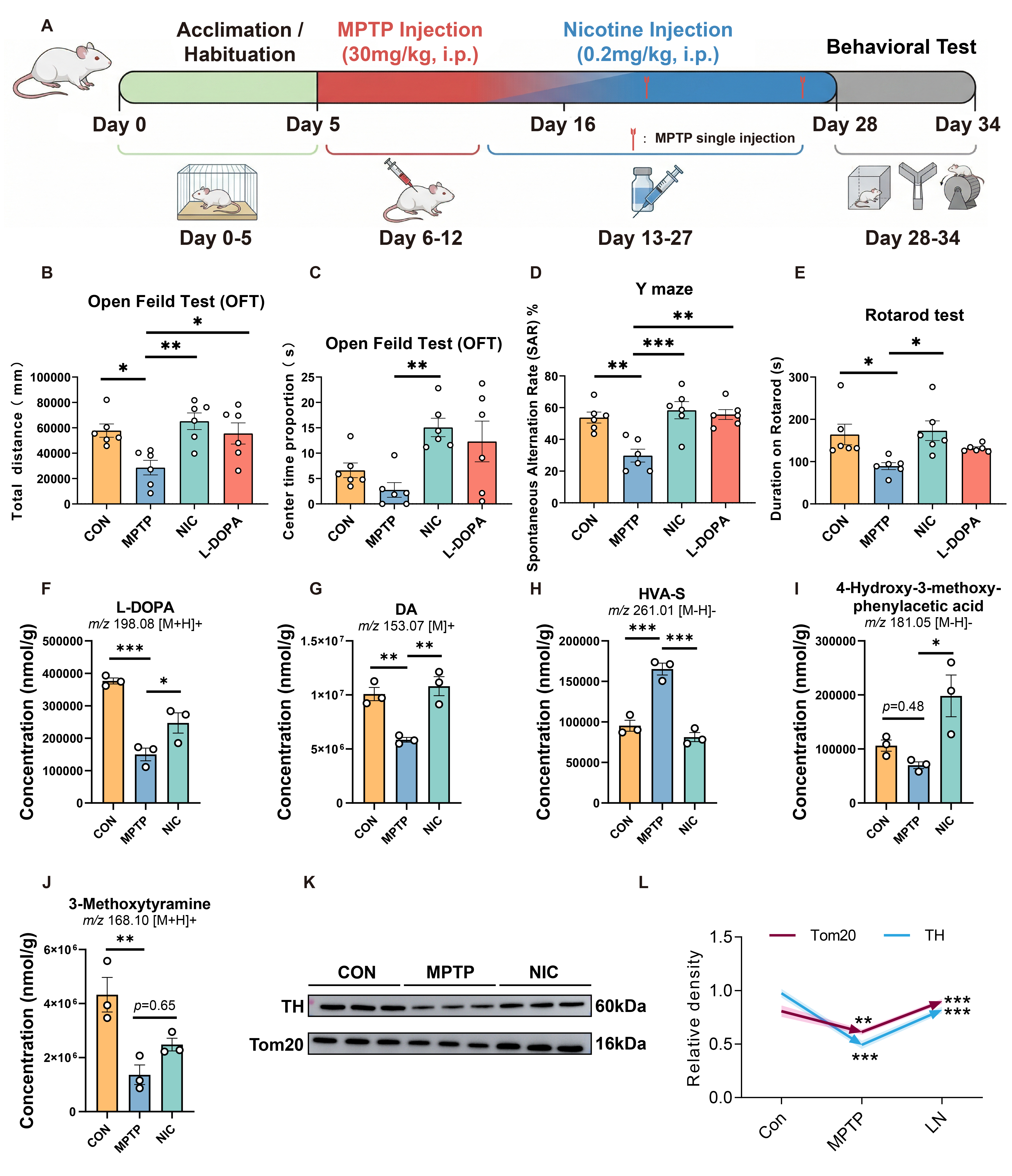

Supplement: Supplementary file 3 [file Image1.tif]

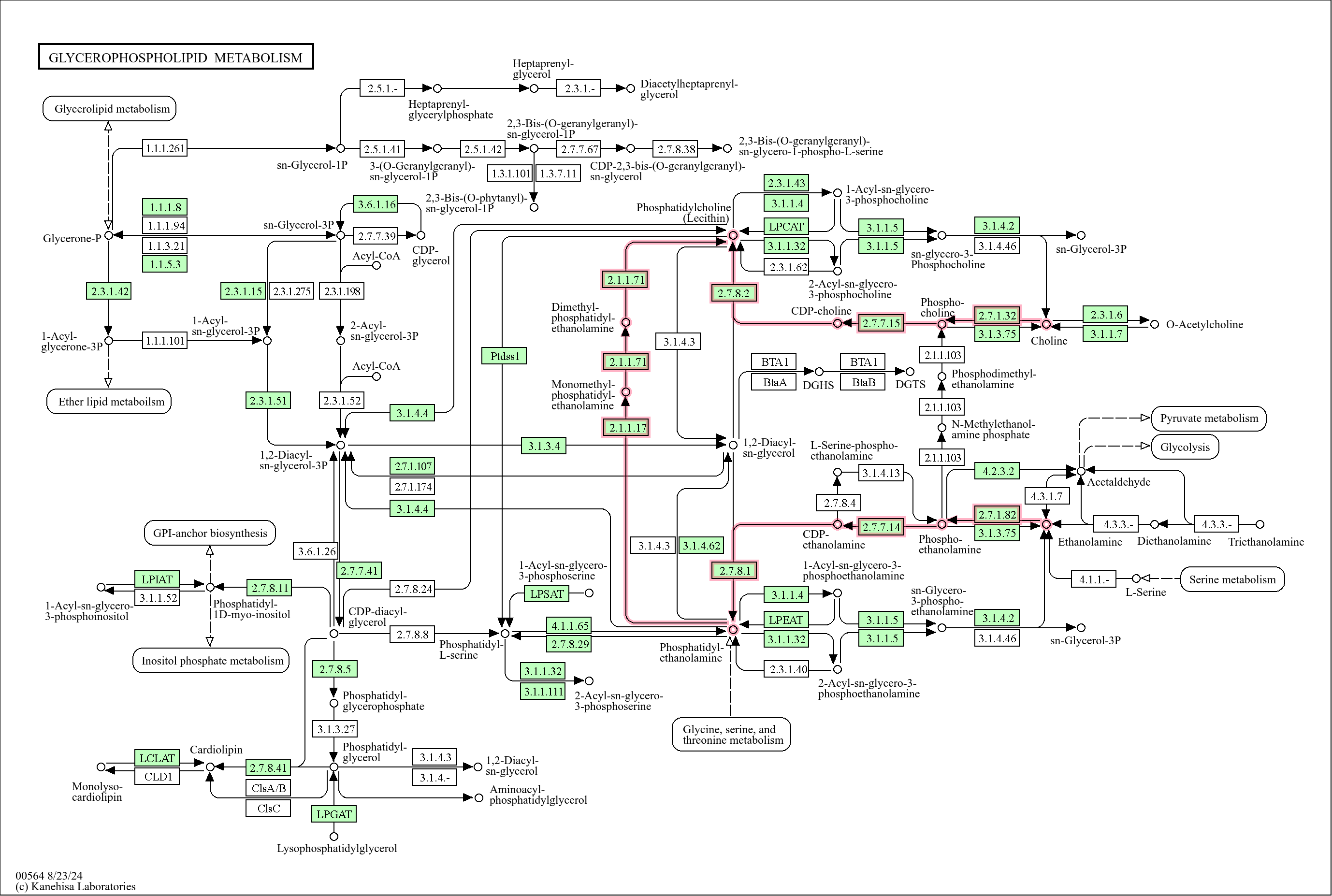

Supplement: Supplementary file 4 [file Image4.png]
