# Supplementary material for: Exploring In situ neuroprotective mechanisms of nicotine in an MPTP-Induced Parkinson’s disease rat model using spatial metabolomics
Source: Front Cell Dev Biol. 2026 May 1;14:1818201. doi: 10.3389/fcell.2026.1818201 (PMC13175961; doi:10.3389/fcell.2026.1818201)
Supplement: Supplementary file 6 [file DataSheet1.docx]

**Supplementary Materials**

**Supplementary Fig. S1.** Nicotine improves behavioral deficits and regulates systemic neurochemical levels in MPTP-induced Parkinson's disease (PD) rats.

**Supplementary Fig. S2.** Glycerophospholipid metabolism and mitochondrial redox indicators in response to nicotine injection.

**Supplementary Fig. S3.** Mass Spectra of Representative Ions.

**Supplementary Fig. S4.** Schematic Diagram of Nicotine-Mediated Regulatory Mechanisms of the Glycerophospholipid Metabolism Pathway.

**Supplementary Table S1.** Significantly Differentially Expressed Metabolites (Top 10) in Six Key Brain Regions.

**Supplementary Table S2.** Source data for bar plots shown in the main text, organized by brain region (STR, HIPP, THA, HYP, PIN, and MB).

**Supplementary Table S3.** LC-MS/MS quantitative data for validated metabolites.

**Exploring *In Situ* Neuroprotective Mechanisms of Nicotine in an MPTP-Induced Parkinson’s Disease Rat Model Using Spatial Metabolomics**

**Fig. S1. Nicotine improves behavioral deficits and regulates systemic neurochemical levels in MPTP-induced Parkinson's disease (PD) rats.**


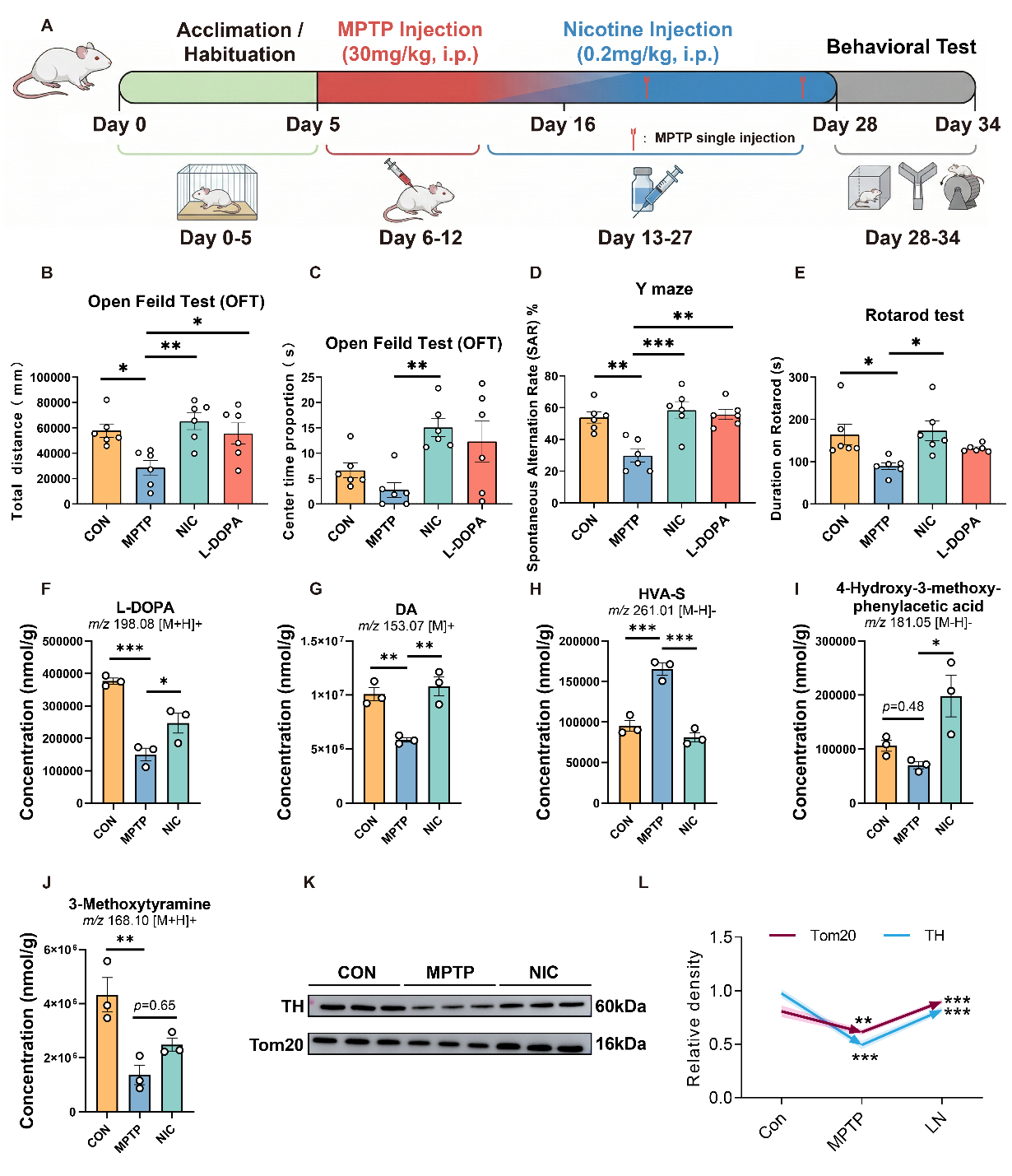


**Fig. S1 Nicotine ameliorates behavioral deficits and restores neurotransmitter and mitochondrial protein levels in MPTP-induced PD rats. (A)** Experimental timeline depicting acclimation/habituation (Day 0–5), MPTP administration (30 mg/kg, *i.p.*; Day 6–12), nicotine treatment (0.2 mg/kg, *i.p.*; Day 13–27), and behavioral tests (Day 28–34). MPTP single injection indicated by red arrow. **(B–E)** Behavioral assessment results: **(B)** total distance traveled and **(C)** center time in the Open Field Test (OFT), **(D)** spontaneous alternation rate (SAR) in the Y-maze, and **(E)** fall latency in the Rotarod test. Data indicate that MPTP induces locomotor, cognitive, and motor coordination deficits, which are significantly ameliorated by nicotine. **(F–J)** LC-MS/MS quantification of striatal neurotransmitters: **(F)** L-DOPA, **(G)** dopamine (DA), **(H)** homovanillic acid sulfate (HVA-S), **(I)** 4-hydroxy-3-methoxyphenylacetic acid, and **(J)** 3-methoxytyramine. Nicotine treatment significantly restores DA metabolism disrupted by MPTP. **(K–L)** Western blot analysis of tyrosine hydroxylase (TH) and mitochondrial marker Tom20 in striatal tissue. **(K)** Representative blots. **(L)** Quantification of relative protein density normalized to Tom20. Nicotine rescues MPTP-induced reduction of TH and partially restores mitochondrial protein levels. Data are presented as mean ± SEM (n=4–6 per group). Statistical significance was determined using one-way ANOVA followed by Tukey’s post hoc test: **P<0.05, **P<0.01, ***P<0.001*.

**Fig. S2. Glycerophospholipid metabolism and mitochondrial redox indicators in response to nicotine injection.**

**
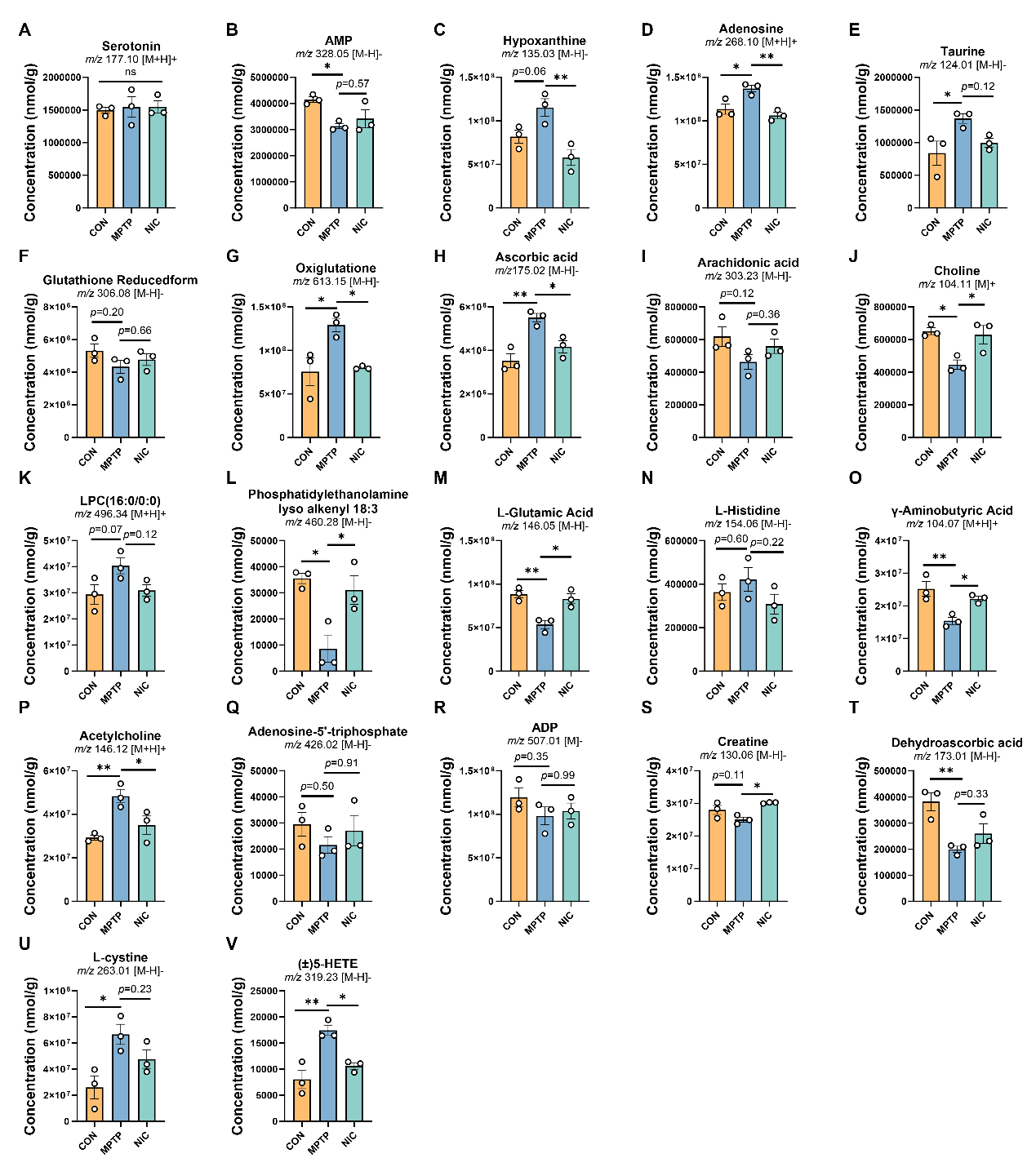
**

**Fig.S2 Glycerophospholipid metabolism and mitochondrial redox indicators in response to nicotine injection. (A–V)** LC-MS/MS quantification of metabolites associated with energy metabolism and oxidative stress, including **(A)** serotonin, **(B)** AMP, **(C)** hypoxanthine, **(D)** adenosine, **(E)** taurine, **(F)** reduced glutathione (GSH), **(G)** oxidized glutathione (GSSG), **(H)** ascorbic acid, **(I)** arachidonic acid, **(J)** choline, **(K)** LPC(16:0/0:0), **(L)** phosphatidylethanolamine lyso alkenyl 18:3, **(M)** L-glutamic acid, **(N)** L-histidine, **(O)** GABA, **(P)** acetylcholine, **(Q)** ATP, **(R)** ADP, **(S)** creatine, **(T)** dehydroascorbic acid, **(U)** L-cystine, **(V)** (±)-5-HETE. Nicotine significantly reverses MPTP-induced alterations in mitochondrial redox balance and lipid-related metabolites. Statistical significance of metabolite changes was evaluated using one-way ANOVA with Tukey’s post hoc test: **P<0.05, **P<0.01*.

.

**Fig. S3. Mass Spectra of Representative Ions.**


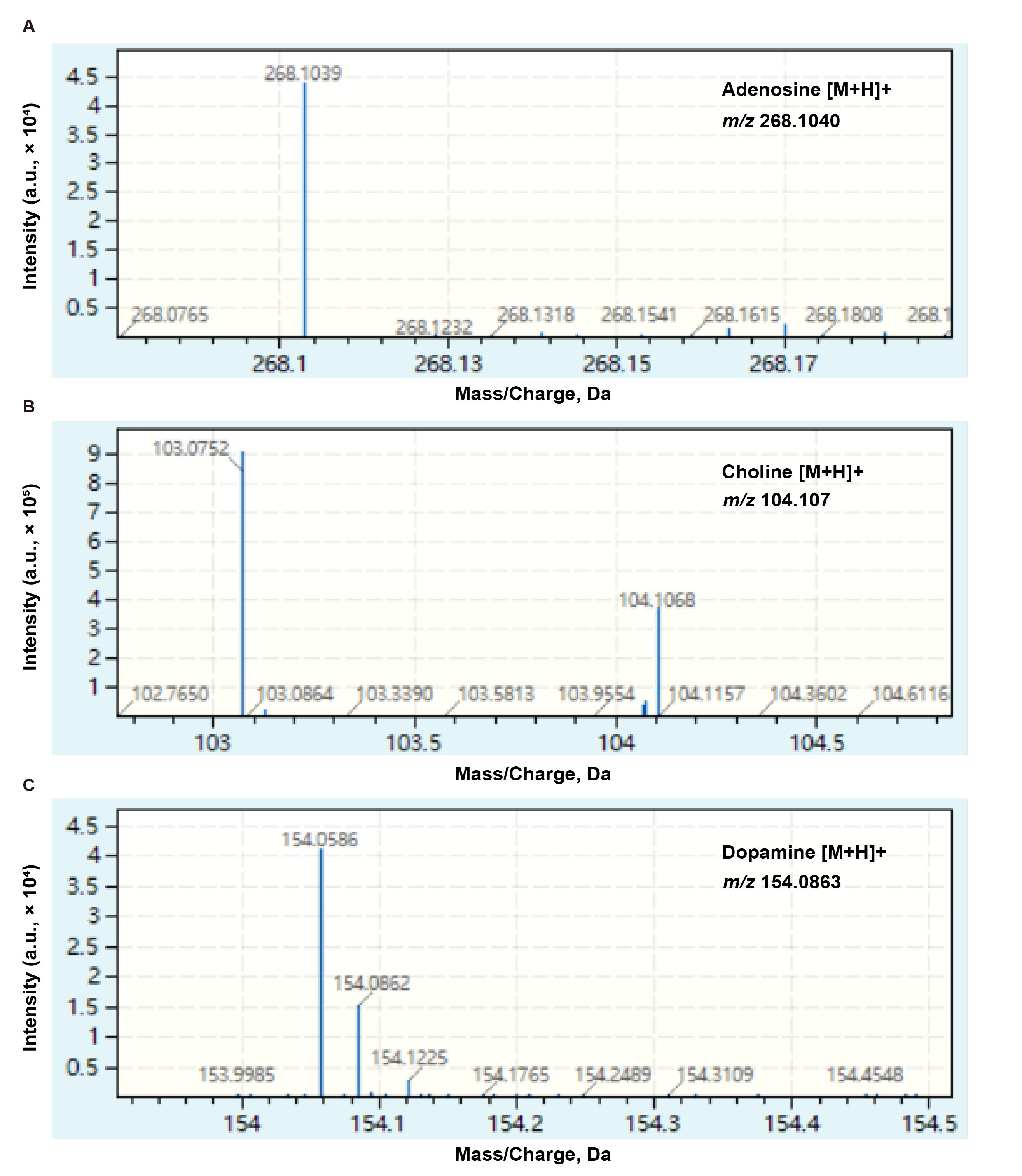


**Fig. S3 Mass Spectra of Representative Ions. A**. Adenosine ([M+H]⁺, m/z 268.1040). **B**. Choline ([M+H]⁺, m/z 104.1070). **C**. Dopamine ([M+H]⁺, m/z 154.0863). High-resolution mass spectrometry confirmed the structural identification of the visualized target ions in the MSI analysis.

**Fig. S4. Schematic Diagram of Nicotine-Mediated Regulatory Mechanisms of the Glycerophospholipid Metabolism Pathway.**


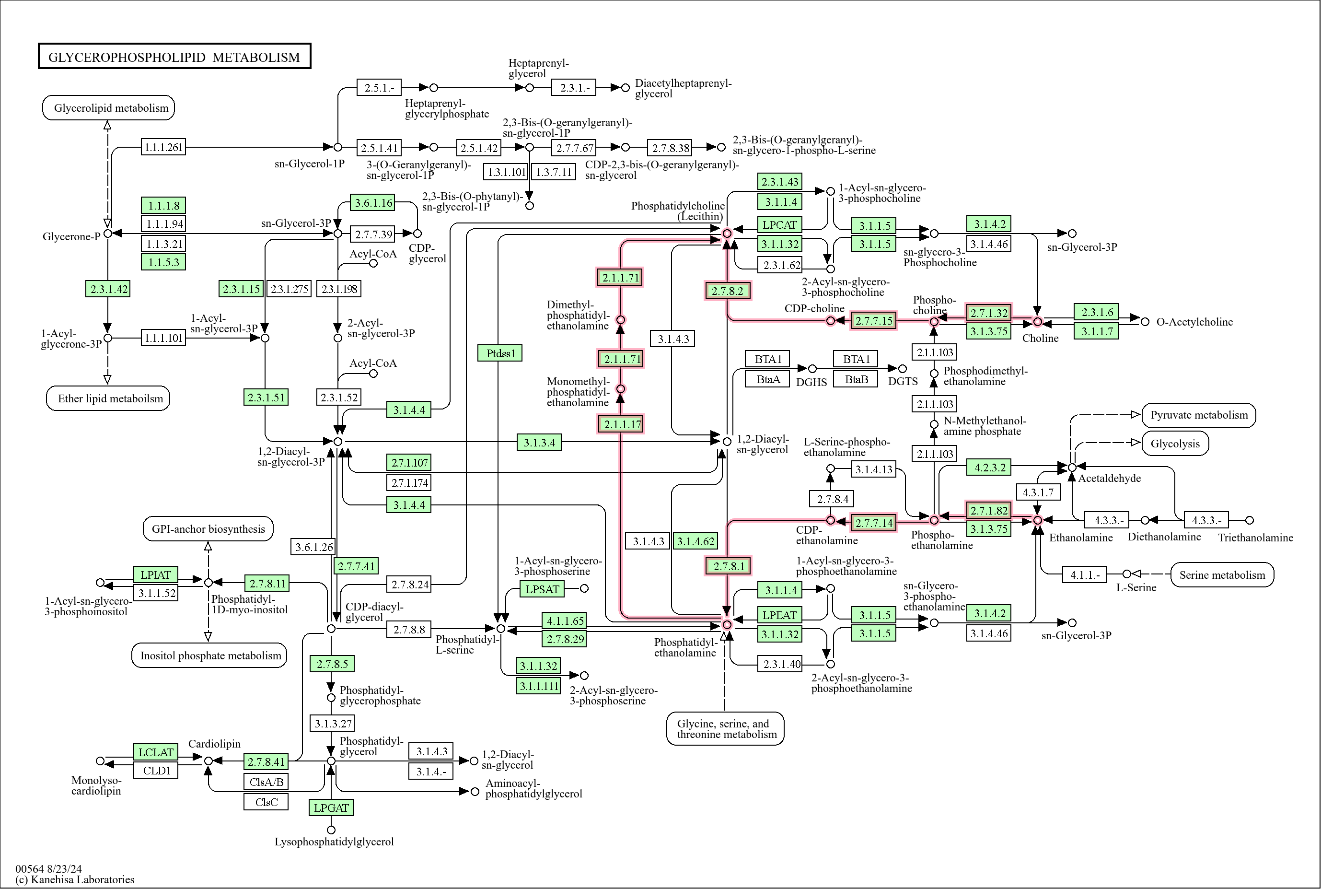


**Fig. S4 Mapping of Differential Metabolites in the KEGG Glycerophospholipid Metabolism Pathway (rno00564).** This pathway diagram illustrates the complex metabolic network involving key lipid molecules and neurotransmitter precursors regulated by MPTP and nicotine. Key nodes highlight the metabolic conversion between key components maintaining membrane integrity (phosphatidylcholine (PC) and phosphatidylethanolamine (PE)), as well as the degradation pathway leading to the production of lysophosphatidylcholine (LysoPC). Notably, this pathway tightly links membrane lipid metabolism to the cholinergic system via choline and acetylcholine (right), providing direct bioinformatics evidence that nicotine exerts neuroprotective effects by synergistically repairing membrane lipid homeostasis and restoring cholinergic neurotransmitter precursors. Green boxes indicate enzymes associated with matched metabolites in the enrichment analysis.

**Table S1. Significantly Differentially Expressed Metabolites (Top 10) in Six Key Brain Regions.**

| No. | Encephalic region | m/z | Formula | Adduct | VIP | FC | *P*-Value | Identification | class |
| --- | --- | --- | --- | --- | --- | --- | --- | --- | --- |
| 1 | STR | 160.0968 | C7H13NO3 | [M+H]+ | 1.748678135 | 0.02400557 | 0.015993251 | Calystegin A3 | Others |
| 2 | STR | 164.1394 | C6H14N2O2 | [M+NH4]+ | 1.641297962 | 9.510143986 | 0.039132948 | L-Lysine | Amino acids |
| 3 | STR | 309.2213 | C22H32O2 | [M-H-H2O]- | 1.526686211 | 0 | 0.044981444 | Docosahexaenoic acid | Fatty Acyls |
| 4 | STR | 756.5538 | C42H78NO8P | [M+H]+ | 1.509660632 | 0.214177483 | 0.02969314 | PC(20:3(8Z,11Z,14Z)/14:0) | Glycerophospholipids |
| 5 | STR | 848.5566 | C46H84NO8P | [M+K]+ | 1.490476349 | 0.248157443 | 0.044556414 | PC(22:4(7Z,10Z,13Z,16Z)/16:0) | Glycerophospholipids |
| 6 | STR | 804.5538 | C46H78NO8P | [M+H]+ | 1.333752757 | 0.252046721 | 0.033066245 | PC(22:6(4Z,7Z,10Z,13Z,16Z,19Z)/16:1(9Z)) | Glycerophospholipids |
| 7 | STR | 800.5565 | C45H80NO7P | [M+Na]+ | 1.159325677 | 0.144535306 | 0.037020453 | PE(P-18:1(11Z)/22:4(7Z,10Z,13Z,16Z)) | Glycerophospholipids |
| 8 | STR | 175.0248 | C6H8O6 | [M-H]- | 1.074329837 | 9.535924455 | 0.040117065 | Ascorbic acid | Others |
| 9 | STR | 814.5357 | C45H78NO8P | [M+Na]+ | 1.019079747 | 0.243088302 | 0.019707942 | PE(18:0/22:6(4Z,7Z,10Z,13Z,16Z,19Z)) | Glycerophospholipids |
| 10 | STR | 724.5276 | C41H74NO7P | [M+H]+ | 1.009301052 | 0.545117794 | 0.044287534 | PE(P-18:1(11Z)/18:3(6Z,9Z,12Z)) | Glycerophospholipids |
| 11 | HIPP | 170.0924 | C7H11N3O2 | [M+H]+ | 1.863681967 | 0 | 0.035816642 | 3-Methylhistidine | Amino acids |
| 12 | HIPP | 792.5538 | C45H78NO8P | [M+H]+ | 1.686952257 | 2.168329064 | 0.044342515 | PE(18:0/22:6(4Z,7Z,10Z,13Z,16Z,19Z)) | Glycerophospholipids |
| 13 | HIPP | 168.1019 | C9H13NO2 | [M+H]+ | 1.686508441 | 0 | 0.04447266 | 4-Methoxytyramine | Others |
| 14 | HIPP | 146.1175 | C7H15NO2 | [M+H]+ | 1.650769424 | 0 | 0.002036431 | 3-Dehydroxycarnitine | Fatty Acyls |
| 15 | HIPP | 783.5647 | C43H76NO8P | [M+NH4]+ | 1.514597192 | 0.538030558 | 0.021924105 | PE(22:5(4Z,7Z,10Z,13Z,16Z)/16:0) | Glycerophospholipids |
| 16 | HIPP | 281.2486 | C18H34O2 | [M-H]- | 1.339935803 | 4.214039701 | 0.014271435 | Oleic acid | Fatty Acyls |
| 17 | HIPP | 309.2799 | C20H38O2 | [M-H]- | 1.129771509 | 16.93276263 | 0.000247062 | 11Z-Eicosenoic acid | Fatty Acyls |
| 18 | HIPP | 724.5276 | C41H74NO7P | [M+H]+ | 1.082477983 | 0.68953286 | 0.046042127 | PE(P-18:1(11Z)/18:3(6Z,9Z,12Z)) | Glycerophospholipids |
| 19 | HIPP | 303.233 | C20H32O2 | [M-H]- | 1.035264564 | 3.302300879 | 0.036432257 | Arachidonic acid | Fatty Acyls |
| 20 | HIPP | 218.1034 | C9H17NO5 | [M-H]- | 0.909525986 | 4.256981911 | 0.015752193 | Pantothenic acid | Others |
| 21 | THA | 261.0202 | C9H18S3 | [M+K]+ | 1.857432691 | 0 | 0.009026994 | 2,2,4,4,6,6-Hexamethyl-1,3,5-trithiane | Others |
| 22 | THA | 275.1366 | C8H20NO6P | [M+NH4]+ | 1.80759441 | 99999 | 0.009242732 | Glycerophosphocholine | Glycerophospholipids |
| 23 | THA | 834.6007 | C48H84NO8P | [M+H]+ | 1.917889946 | 0 | 0.009499821 | PC(20:2(11Z,14Z)/20:4(5Z,8Z,11Z,14Z)) | Glycerophospholipids |
| 24 | THA | 427.1024 | C22H18O9 | [M+H]+ | 1.800744697 | 11097.75958 | 0.009745619 | (-)-Epiafzelechin 3-gallate | Others |
| 25 | THA | 792.5538 | C45H78NO8P | [M+H]+ | 1.176044017 | 1.470658488 | 0.013938179 | PE(18:0/22:6(4Z,7Z,10Z,13Z,16Z,19Z)) | Glycerophospholipids |
| 26 | THA | 806.5694 | C46H80NO8P | [M+H]+ | 1.560003362 | 0.492659695 | 0.014727576 | PC(22:5(7Z,10Z,13Z,16Z,19Z)/16:1(9Z)) | Glycerophospholipids |
| 27 | THA | 247.1012 | C12H16N4OS | [M+H-H2O]+ | 1.825997555 | 99999 | 0.015993529 | Thiamine | Others |
| 28 | THA | 783.5647 | C43H76NO8P | [M+NH4]+ | 1.236086779 | 0.525941548 | 0.016908487 | PE(22:5(4Z,7Z,10Z,13Z,16Z)/16:0) | Glycerophospholipids |
| 29 | THA | 283.2642 | C18H36O2 | [M-H]- | 1.507455823 | 1220.47441 | 0.020000061 | Ethyl hexadecanoate | Fatty Acyls |
| 30 | THA | 186.0873 | C7H13N3O4 | [M+H-H2O]+ | 1.24273578 | 3.295561434 | 0.037248342 | Glutaminylglycine | Amino acids |
| 31 | HYP | 104.107 | C5H13NO | [M+H]+ | 1.65533806 | 0.110467104 | 0.020621494 | Choline | Others |
| 32 | HYP | 296.066 | C8H20NO6P | [M+K]+ | 1.077577682 | 0.237341234 | 0.049146107 | Glycerophosphocholine | Glycerophospholipids |
| 33 | HYP | 307.2642 | C20H36O2 | [M-H]- | 1.507171059 | 99999 | 0.033929604 | Eicosadienoic acid | Fatty Acyls |
| 34 | HYP | 534.2956 | C24H50NO7P | [M+K]+ | 1.442633056 | 0.16739569 | 0.036647612 | LysoPC(16:0) | Glycerophospholipids |
| 35 | HYP | 331.2642 | C22H36O2 | [M-H]- | 1.340764205 | 3.552563996 | 0.02779462 | Adrenic acid | Fatty Acyls |
| 36 | HYP | 892.5253 | C50H80NO8P | [M+K]+ | 1.359430286 | 5.289651681 | 0.044904557 | PC(22:5(4Z,7Z,10Z,13Z,16Z)/20:5(5Z,8Z,11Z,14Z,17Z)) | Glycerophospholipids |
| 37 | HYP | 148.0393 | C8H9NO3 | [M-H-H2O]- | 1.772777938 | 0.017483694 | 0.025208237 | Pyridoxal | Others |
| 38 | HYP | 328.0442 | C10H14N5O7P | [M-H-H2O]- | 1.061017042 | 3.42914765 | 0.032815553 | 3'-AMP | Nucleosides & Nucleotides |
| 39 | HYP | 332.0952 | C11H19NO9 | [M+Na]+ | 1.596724498 | 0.176296313 | 0.02573126 | N-Acetylneuraminic acid | Others |
| 40 | HYP | 148.0393 | C8H5NO2 | [M+H]+ | 1.752055132 | 0.015266581 | 0.01972012 | 1H-Indole-2,3-dione | Others |
| 41 | PIN | 872.5566 | C48H84NO8P | [M+K]+ | 1.547075376 | 0.026368812 | 0.010284565 | PC(20:2(11Z,14Z)/20:4(5Z,8Z,11Z,14Z)) | Glycerophospholipids |
| 42 | PIN | 798.541 | C42H82NO8P | [M+K]+ | 1.28905006 | 0.197469516 | 0.028919646 | PC(20:1(11Z)/14:0) | Glycerophospholipids |
| 43 | PIN | 844.5253 | C46H80NO8P | [M+K]+ | 1.27903007 | 0.113733691 | 0.00519695 | PC(22:5(7Z,10Z,13Z,16Z,19Z)/16:1(9Z)) | Glycerophospholipids |
| 44 | PIN | 152.0471 | C4H12NO4P | [M+H-H2O]+ | 1.19631786 | 0.163858636 | 0.037179858 | Phosphodimethylethanolamine | Others |
| 45 | PIN | 152.0221 | C4H7N3O | [M+K]+ | 1.31569569 | 0.129701567 | 0.016041746 | Creatinine | Amino acids |
| 46 | PIN | 170.0326 | C4H9N3O2 | [M+K]+ | 1.346242444 | 0.139664379 | 0.022564541 | Creatine | Amino acids |
| 47 | PIN | 206.1499 | C8H16N2O3 | [M+NH4]+ | 1.577167685 | 6.685257233 | 0.011715994 | N-Alpha-acetyllysine | Amino acids |
| 48 | PIN | 135.0917 | C8H7N | [M+NH4]+ | 1.625028711 | 8.257638053 | 0.031946822 | Indole | Others |
| 49 | PIN | 107.0604 | C6H8N2O | [M+H-H2O]+ | 1.663906027 | 5.360714723 | 0.039496121 | Methylimidazole acetaldehyde | Others |
| 50 | PIN | 103.0866 | C4H7NO | [M+NH4]+ | 1.625867052 | 8.380463484 | 0.039473782 | 2-Pyrrolidinone | Others |
| 51 | MB | 170.0924 | C7H11N3O2 | [M+H]+ | 1.754727501 | 0 | 0.034733042 | 3-Methylhistidine | Amino acids |
| 52 | MB | 131.058 | C6H8N2 | [M+Na]+ | 1.841787829 | 0 | 0.03095882 | 2,5-Dimethylpyrazine | Others |
| 53 | MB | 160.0968 | C7H13NO3 | [M+H]+ | 1.844066358 | 0 | 0.001584812 | Calystegin A3 | Others |
| 54 | MB | 227.0662 | C9H12N2O6 | [M+H-H2O]+ | 1.484078768 | 0.000285043 | 0.00748749 | Uridine | Nucleosides & Nucleotides |
| 55 | MB | 786.6007 | C44H84NO8P | [M+H]+ | 1.416263774 | 0.170297518 | 0.022628707 | PC(22:2(13Z,16Z)/14:0) | Glycerophospholipids |
| 56 | MB | 804.5538 | C46H78NO8P | [M+H]+ | 1.598875744 | 0.17097683 | 0.025702376 | PC(22:6(4Z,7Z,10Z,13Z,16Z,19Z)/16:1(9Z)) | Glycerophospholipids |
| 57 | MB | 821.5803 | C46H78NO8P | [M+NH4]+ | 1.562484102 | 0.194897044 | 0.027889629 | PC(22:6(4Z,7Z,10Z,13Z,16Z,19Z)/16:1(9Z)) | Glycerophospholipids |
| 58 | MB | 826.5723 | C44H86NO8P | [M+K]+ | 1.363884079 | 0.243720125 | 0.04640433 | PC(18:1(9Z)/18:0) | Glycerophospholipids |
| 59 | MB | 832.5851 | C48H82NO8P | [M+H]+ | 1.542571392 | 0.245254759 | 0.030808251 | PC(20:4(8Z,11Z,14Z,17Z)/20:3(8Z,11Z,14Z)) | Glycerophospholipids |
| 60 | MB | 214.0488 | C6H13NO5 | [M+Cl]- | 1.36483807 | 99999 | 0.038217137 | beta-D-Glucosamine | Carbohydrates |

Identification information and statistical analysis of potential biomarkers. Abbreviations: STR, striatum; HIPP, hippocampus; THA, thalamus; HYP, hypothalamus; PIN, pineal gland; MB, midbrain; VIP, Variable Importance in Projection; FC, fold change (MPTP/CON or NIC/MPTP).
